# Acute Aromatase Inhibition Impairs Neural and Behavioral Auditory Scene Analysis in Zebra Finches

**DOI:** 10.1523/ENEURO.0423-23.2024

**Published:** 2024-03-20

**Authors:** Marcela Fernández-Vargas, Matheus Macedo-Lima, Luke Remage-Healey

**Affiliations:** Neuroscience and Behavior Program, Center for Neuroendocrine Studies, University of Massachusetts Amherst, Amherst, Massachusetts 01003

**Keywords:** aromatase inhibition, auditory scene analysis, caudomedial nidopallium, extracellular recordings, operant conditioning, zebra finches

## Abstract

Auditory perception can be significantly disrupted by noise. To discriminate sounds from noise, auditory scene analysis (ASA) extracts the functionally relevant sounds from acoustic input. The zebra finch communicates in noisy environments. Neurons in their secondary auditory pallial cortex (caudomedial nidopallium, NCM) can encode song from background chorus, or scenes, and this capacity may aid behavioral ASA. Furthermore, song processing is modulated by the rapid synthesis of neuroestrogens when hearing conspecific song. To examine whether neuroestrogens support neural and behavioral ASA in both sexes, we retrodialyzed fadrozole (aromatase inhibitor, FAD) and recorded in vivo awake extracellular NCM responses to songs and scenes. We found that FAD affected neural encoding of songs by decreasing responsiveness and timing reliability in inhibitory (narrow-spiking), but not in excitatory (broad-spiking) neurons. Congruently, FAD decreased neural encoding of songs in scenes for both cell types, particularly in females. Behaviorally, we trained birds using operant conditioning and tested their ability to detect songs in scenes after administering FAD orally or injected bilaterally into NCM. Oral FAD increased response bias and decreased correct rejections in females, but not in males. FAD in NCM did not affect performance. Thus, FAD in the NCM impaired neuronal ASA but that did not lead to behavioral disruption suggesting the existence of resilience or compensatory responses. Moreover, impaired performance after systemic FAD suggests involvement of other aromatase-rich networks outside the auditory pathway in ASA. This work highlights how transient estrogen synthesis disruption can modulate higher-order processing in an animal model of vocal communication.

## Significance Statement

The ability to extract relevant sounds from a complex acoustic input, or acoustic scene, can decline with age and is often impaired in auditory and neurological disorders. Moreover, hearing decline and speech-in-noise processing have been associated with hormonal changes during perimenopause or menopause in women. However, hormone-dependent hearing impairments are understudied in female animal models. Here, we explore the role of neuroestrogen synthesis in auditory scene analysis (ASA) in a songbird. We found that blocking the synthesis of neuroestrogens with the drug fadrozole (FAD) impaired ASA in auditory neurons. FAD administered orally caused females to decrease correct rejections and respond indiscriminately during behavioral ASA using operant conditioning. These results elucidate potential neural and hormonal mechanisms underlying speech-in-noise impairments in women.

## Introduction

Auditory perception and social communication can be significantly disrupted by background noise (Brumm, 2013). To discriminate sounds from noise, the brain must detect functionally relevant sounds present in the auditory input. This perceptual capability is known as auditory scene analysis (ASA) (Hulse, 2002; Shamma, 2008) and it can deteriorate with age or be impaired in some brain disorders (Lin et al., 2017; Hardy et al., 2020; Helfer and Jesse, 2020; Maggu and Overath, 2021). Despite its relevance in auditory function, much of the physiological underpinnings remains unknown (Itatani and Klump, 2017; Snyder and Elhilali, 2017).

**Table 1.** Statistical table

Experiment	Dependent variable	Test	Factors: results	Post hoc	Fig.
**Retrodialysis treatment control (PRE) vs FAD—extracellular recordings**					
NS in response to 63 dB song	Log (zeromin (*z*-scores))	GLMM	Treatment: *X*^2^_(1)_ = 12.41, *p* = 0.000426 Sex: *X*^2^_(1)_ = 0.20, *p* = 0.65 Treatment × Sex: *X*^2^_(1)_ = 0.04, *p* = 0.833		2e
BS in response to 63 dB song	Sqrt (zeromin (*z*-scores))	GLMM	Treatment: *X*^2^_(1)_ = 3.55, *p* = 0.059 Sex: *X*^2^_(1)_ = 2.13, *p* = 0.14 Treatment × Sex: *X*^2^_(1)_ = 1.74, *p* = 0.187		2f
NS in response to 48–78 dB songs	Log (% classifier accuracy)	GLMM	Treatment: *X*^2^_(1)_ = 4.5, *p* = 0.034 Sex: *X*^2^_(1)_ = 4.49, *p* = 0.034 Treatment × Sex: *X*^2^_(1)_ = 3.33, *p* = 0.068		3c
BS in response to 48–78 dB songs	Log (% classifier) accuracy	GLMM	Treatment: *X*^2^_(1)_ = 1.32, *p* = 0.251 Sex: *X*^2^_(1)_ = 0.013, *p* = 0.91 Treatment × Sex: *X*^2^_(1)_ = 1.20, *p* = 0.273		3d
NS in response to 63 dB song	Latency (s)	GLMM	Treatment: *X*^2^_(1)_ = 0.878, *p* = 0.349, Sex: *X*^2^_(1)_ = 0.000, *p* = 0.986, Treatment × Sex: *X*^2^_(1)_ = 0.016, *p* = 0.900		3e
BS in response to 63 dB song	Latency (s)	GLMM	Treatment: *X*^2^_(1)_ = 0.302, *p* = 0.583, Sex: *X*^2^_(1)_ = 1.466, *p* = 0.226, Treatment × Sex: *X*^2^_(1)_ = 0.064, *p* = 0.800		3f
NS in response to scenes (−15 to 15 dB SNR)	Sqrt (zeromin (Neural threshold, dB SNR)	GLMM	Treatment: *X*^2^_(1)_ = 5.87, *p* = 0.015, Sex: *X*^2^_(1)_ = 4.17, *p* = 0.041, Treatment × Sex, *X*^2^_(1)_ = 23.4, *p* *<* 0.001	Female PRE-FAD vs FAD: *t*_(18)_ = 5.19, *p* = 0.001, male PRE-FAD vs FAD: *t*_(18)_ = 1.5, *p* = 0.15 PRE female vs male: *t*_(18)_ = 4.86, *p* = 0.001, FAD female- vs male: *t*_(18)_ = 1.97, *p* = 0.0654	5d
BS in response to scenes (−15 to 15 dB SNR)	Neural threshold, dB SNR	GLMM	Treatment: *X*^2^_(1)_ = 9.47, *p* = 0.0021 Sex: *X*^2^_(1)_ = 0.18, *p* = 0.67, Treatment × Sex: *X*^2^_(1)_ = 3.8, *p* = 0.0511		5d
**Oral treatment control (PRE) vs FAD—operant task**					
Discrimination of the 63 dB SPL GO song	% Correct during time window #1 (T1)	Wilcoxon signed-rank test	Males PRE-FAD vs FAD *z* = −1.96, *p* = 0.05, *d* = 0.69 Females PRE-FAD vs FAD *z* = −0.524, *p* = 0.6		6a
	% Correct during time window #1 (T2)	Wilcoxon signed-rank test	Males PRE-FAD vs FAD *z* = −0.7, *p* = 0.48 Females PRE-FAD vs FAD *z* = 0.73, *p* = 0.46		6a
Discrimination of scenes (−15 to 10 dB SNR) during T1	% Correct <0 dB SNR	GLMM	Treatment: *X*^2^_(1)_ = 1.03, *p* = 0.309, Sex: *X*^2^_(1)_ = 13.5, *p* = 0.0002, Treatment × Sex: *X*^2^_(1)_ = 1.93, *p* = 0.16		6b
	% Correct ≥0 dB SNR	GLMM	Treatment: *X*^2^_(1)_ = 0.68, *p* = 0.41, Sex: *X*^2^_(1)_ = 0.0046, *p* = 0.94, Treatment × Sex: *X*^2^_(1)_ = 0.43, *p* = 0.51		6b
	Log (Hit rate) <0 dB SNR	GLMM	Treatment: *X*^2^_(1)_ = 1.95, *p* = 0.16, Sex: *X*^2^_(1)_ = 4.68, *p* = 0.03, Treatment × Sex: *X*^2^_(1)_ = 4.81, *p* = 0.028	Female PRE-FAD vs FAD: *t*_(21)_ = 2.57, *p* = 0.017, male PRE-FAD vs FAD: *t*_(21)_ = 0.38, *p* = 0.71 PRE female vs male: *t*_(21)_ = 2.97, *p* = 0.073, FAD female vs male: *t*_(21)_ = 0.78, *p* = 0.44	6c
	Hit rate ≥0 dB SNR	GLMM	Treatment: *X*^2^_(1)_ = 0.0, *p* = 0.99, Sex: *X*^2^_(1)_ = 2.85, *p* = 0.09, Treatment × Sex: *X*^2^_(1)_ = 0.87, *p* = 0.35		6c
	Sqrt (Rejection rate) <0 dB SNR	GLMM	Treatment: *X*^2^_(1)_ = 5.71, *p* = 0.017, Sex: *X*^2^_(1)_ = 0.34, *p* = 0.558, Treatment × Sex: *X*^2^_(1)_ = 9.35, *p* = 0.002	Female PRE-FAD vs FAD: *t*_(21)_ = 3.82, *p* = 0.001, male PRE-FAD vs FAD: *t*_(21)_ = 0.19, *p* = 0.85	6d
	Rejection rate ≥0 dB SNR	GLMM	Treatment: *X*^2^_(1)_ = 0.63, *p* = 0.426, Sex: *X*^2^_(1)_ = 2.32, *p* = 0.127, Treatment × Sex: *X*^2^_(1)_ = 3.18, *p* = 0.0745		
	Response bias (c) <0 dB SNR	GLMM	Treatment: *X*^2^_(1)_ = 3.39, *p* = 0.06, Sex: *X*^2^_(1)_ = 1.09, *p* = 0.295, Treatment × Sex: *X*^2^_(1)_ = 8.10, *p* = 0.004	Female PRE-FAD vs FAD: *t*_(21)_ = 3.35, *p* = 0.003, male PRE-FAD vs FAD: *t*_(21)_ = 0.47, *p* = 0.64 PRE female vs male: *t*_(21)_ = 2.97, *p* = 0.032, FAD female vs male: *t*_(21)_ = 0.46, *p* = 0.64	6e
	Response bias (c) ≥0 dB SNR	GLMM	Treatment: *X*^2^_(1)_ = 0.12, *p* = 0.73, Sex: *X*^2^_(1)_ = 2.66, *p* = 0.102, Treatment × Sex: *X*^2^_(1)_ = 2.33, *p* = 0.13		6e
	Number of trials	Wilcoxon signed-rank test	Females: *z* = 1.15, *p* = 0.249 Males: *z* = 0.98, *p* = 0.327		
	Log (Response times) for GO Log (Response times) for NOGO	LMM LMM	Treatment: *F*_(1,20.17)_ = 0.26, *p* = 0.615, Sex: *F*_(1, 20.17)_ = 4.09, *p* = 0.057 Treatment × sex: *F*_(1,20.17)_ = 1.218, *p* = 0.283) Treatment: *F*_(1,22.7)_ = 0.033, *p* = 0.858, Sex: *F*_(1, 22.7)_ = 4.76, *p* = 0.04 Treatment × sex: *F*_(1,22.7)_ = 0.72, *p* = 0.404		
**NCM treatment control (PRE) vs FAD- operant task**					
Discrimination of the 63 dB SPL GO song	% Correct during time window #1 (T1)	Wilcoxon signed-rank test	Males *z* = −1.48, *p* = 0.138 Females *z* = 1.46, *p* = 0.144		7a
	% Correct during time window #1 (T2)	Wilcoxon signed-rank test	Males *z* = 0.135, *p* = 0.89 Females *z* = 0.73, *p* = 0.465		
Discrimination of scenes (−15 to 10 dB SNR) during T1	% Correct <0 dB SNR	GLMM	Treatment: *X*^2^_(1)_ = 0.05, *p* = 0.815, Sex: *X*^2^_(1)_ = 0.91, *p* = 0.338, Treatment × Sex: *X*^2^_(1)_ = 3.62, *p* = 0.057		7b
	% Correct ≥0 dB SNR	GLMM	Treatment: *X*^2^_(1)_ = 2.86, *p* = 0.09, Sex: *X*^2^_(1)_ = 0.008, *p* = 0.93, Treatment × Sex: *X*^2^_(1)_ = 6.87, *p* = 0.009;	Female PRE-FAD vs FAD: *t*_(12)_ = −3.08, *p* = 0.009, male PRE-FAD vs FAD: *t*_(12)_ = 0.49, *p* = 0.63	7b
	Hit rate <0 dB SNR	GLMM	Treatment: *X*^2^_(1)_ = 1.26, *p* = 0.26, Sex: *X*^2^_(1)_ = 3.49, *p* = 0.061, Treatment × Sex: *X*^2^_(1)_ = 3.31, *p* = 0.128		7c
	Hit rate ≥0 dB SNR	GLMM	Treatment: *X*^2^_(1)_ = 1.90, *p* = 0.167, Sex: *X*^2^_(1)_ = 2.35, *p* = 0.125, Treatment × Sex: *X*^2^_(1)_ = 1.5, *p* = 0.22		7c
	Rejection rate <0 dB SNR	GLMM	Treatment: *X*^2^_(1)_ = 0.88, *p* = 0.346, Sex: *X*^2^_(1)_ = 3.79, *p* = 0.0515, Treatment × Sex: *X*^2^_(1)_ = 1.85, *p* = 0.17		7d
	Rejection rate ≥0 dB SNR	GLMM	Treatment: *X*^2^_(1)_ = 0.44, *p* = 0.5, Sex: *X*^2^_(1)_ = 3.29, *p* = 0.069, Treatment × Sex: *X*^2^_(1)_ = 0.01, *p* = 0.91		7d
	Response bias (c) <0 dB SNR	GLMM	Treatment: *X*^2^_(1)_ = 0.38, *p* = 0.53, Sex: *X*^2^_(1)_ = 6.28, *p* = 0.012, Treatment × Sex: *X*^2^_(1)_ = 1.74, *p* = 0.19		7e
	Response bias (c) ≥0 dB SNR	GLMM	Treatment: *X*^2^_(1)_ = 0.77, *p* = 0.38, Sex: *X*^2^_(1)_ = 2.81, *p* = 0.09, Treatment × Sex: *X*^2^_(1)_ = 0.13, *p* = 0.77		7e
	Number of trials	LMM	Treatment: *F*_(1,11.6)_ = 1.32, *p* = 0.273, Sex: *F*_(1, 11.6)_ = 3.95, *p* = 0.071, Treatment × Sex: *F*_(1,11.6)_ = 0.11, *p* = 0.746		
	Log (Response times) for GO Log (Response times) for NOGO	LMM LMM	Treatment: *F*_(1,11.3)_ = 0.005, *p* = 0.94, Sex: *F*_(1, 11.3)_ = 3.88, *p* = 0.074 Treatment × Sex: *F*_(1,11.3)_ = 0.248, *p* = 0.628 Treatment: *F*_(1,12)_ = 0.024, *p* = 0.879, Sex: *F*_(1, 12)_ = 2.3, *p* = 0.154 Treatment × Sex: *F*_(1,12)_ = 0.01, *p* = 0.923		

FAD, fadrozole; NS, narrow spiking neurons; BS, broad spiking neurons; SNR, signal-to-noise-ratio; zeromin, [value +2 × min|value|]. GLMM in R used Type II Wald *X*^2^ tests for fixed effects. LMM in SPSS used Type III *F* tests for fixed effects.

Behaviorally relevant sounds can provide a deeper understanding of ASA (Dooling and Blumenrath, 2013; Theunissen and Elie, 2014; Snyder and Elhilali, 2017). Among nonhuman animals, songbirds stand out for being proficient at ASA (Dooling and Blumenrath, 2013). Behavioral studies using operant-conditioning paradigms have shown that zebra finches (*Taeniopygia guttata*) can discriminate a target song that is occluded by song/chorus mixtures or maskers (Benney and Braaten, 2000; Narayan et al., 2007; Schneider and Woolley, 2013), can resolve spatial unmasking (Dent et al., 2009), and are better at discriminating conspecific than heterospecific vocalizations from auditory scenes (Benney and Braaten, 2000; Lohr et al., 2003). In the auditory system of male zebra finches, ASA emerges in the caudomedial nidopallium (NCM; secondary auditory pallial cortex) with a specific population of neurons that are tolerant to high levels of background noise, referred to as “background-invariant” neurons (Moore et al., 2013; Schneider and Woolley, 2013).

Auditory processing at the level of the NCM is regulated by hormones and substantial evidence shows that estradiol, an abundant estrogen, synthetized from testosterone by the enzyme aromatase, modulates local activation (Vahaba and Remage-Healey, 2018; Remage-Healey, 2020). Both human and zebra finch auditory cortices contain a dense population of neurons that express estrogen receptors and aromatase (Saldanha et al., 2000b; Yague et al., 2006; Azcoitia et al., 2011; Ikeda et al., 2017). Aromatase neurons in the NCM show induction of immediate early genes after song stimulation (Krentzel et al., 2020; de Bournonville et al., 2021; Macedo-Lima et al., 2021). Social interactions and song playback rapidly stimulate aromatase activity and elevate estradiol levels in the NCM of both sexes (Remage-Healey et al., 2008, 2009, 2012). This local estradiol synthesis enhances neural responsiveness and coding within minutes (Remage-Healey et al., 2010, 2012). It is unknown, however, if or how hormonal modulation might support ASA.

Studies examining the estradiol modulation on auditory processing typically employ aromatase inhibitors to block estrogen synthesis. The selective nonsteroidal aromatase inhibitor fadrozole (FAD) has been widely used in the zebra finch. Chronic systemic FAD treatment decreases aromatase activity in both sexes’ telencephalon (Wade et al., 1994) and reduces stimulus-specific adaptation of NCM neurons in males (Yoder et al., 2012). Similarly, acute FAD application in the NCM decreases neural responsiveness to conspecific song (and not heterospecific) in males and females (Remage-Healey et al., 2010). Behaviorally, FAD suppresses typical behavioral male preference for bird's own song versus conspecific song when infused in the left NCM (Remage-Healey et al., 2010). FAD applied systemically or locally in NCM impairs male auditory association learning in an operant task (Macedo-Lima and Remage-Healey, 2020). Finally, acute systemic (via oral or intramuscular administration) FAD can effectively and rapidly reduce estradiol levels and aromatase activity in the NCM (Prior et al., 2014; Alward et al., 2016) and dampens sound evoked activity (ERG1) in the dorsal NCM (Krentzel et al., 2020). Thus, FAD can effectively decrease systemic and local estradiol levels in zebra finches.

Systemic aromatase inhibitors have been associated with negative side effects on brain function in women and in common marmosets (Blaustein, 2017; Gervais et al., 2019). The reduction of circulating estradiol (and other estrogens) induced by menopause has been linked to declines in high frequency hearing and deficits in verbal memory (Greendale et al., 2009; Hederstierna et al., 2010). Therefore, neuroestradiol likely plays a role in auditory function. However, auditory impairments following decline in estradiol levels are largely unexplored in female animal models. Consequently, there is a pressing need to characterize ASA in females and whether it is affected by low estradiol levels to identify potential mechanisms of hormone-dependent hearing dysfunction in women.

Together, these independent lines of inquiry suggest that estradiol may modulate auditory neural response properties and behaviors important for ASA. We hypothesized that blocking estradiol via aromatase inhibition would impair ASA in male and female zebra finches. To test this, we examined whether FAD affects song discrimination in auditory scenes at both neurophysiological and behavioral levels in both sexes.

## Materials and Methods

### Animals

We used male and female adult zebra finches (*Taeniopygia guttata*). Experimental birds were bred and reared in the breeding colony at the University of Massachusetts Amherst. Birds were kept at a 14:10 h light/dark cycle, separated from the breeding colony when they were adults (days post hatch, dph > 120) and housed in single-sex aviaries before being used in an experiment. Birds had food and water *ad libitum*, diet enriched by millet seeds and egg food supplement (Quiko Exotic) and weekly access to water baths. All animal procedures in this study were conducted with approval from the Institutional Animal Care and Use Committee at the University of Massachusetts Amherst.

### Acoustic stimuli

We used zebra finch songs recorded from colonies outside our colony and that are deposited in the zebra finch song database publicly available online (Gardner, 2011). Following the protocol used in Schneider and Woolley (2013), using Adobe Audition, we overlaid seven different songs to construct a chorus background (∼1 s long), applied a time-varying filter for phase-shifting and removed peaks and valleys from the sound envelope (maximum size of phase shift 1,500, filter length 500). The chorus had a duration of 3 s and a sampling rate of 22,050 Hz. The level of the chorus background was adjusted to be 63 dB SPL and was later mixed with songs at different intensity levels to generate auditory scenes.

For song stimuli, we picked four different and unfamiliar songs from the online database that were roughly 2 s in length (containing two motifs and introductory notes, if originally present). For each song, we generated a set of files at different pressure levels (48–78 dB SPL, increment steps of 5 dB). First, the gain of each sound file was normalized to 0 dB re:100% (digital gain) and adjusted to different levels in Audition. Sound pressure (dB SPL) was then adjusted by performing systematic and repeated measurements using a sensitive SPL meter (EXTECH, A-weighted, Slow/Hi mode). The calibration of the SPL meter was tested before every use. After generating the songs at the desired sound level, we mixed them with the 63 dB SPL chorus background. The mixing generated auditory scenes of increasing signal-to-noise ratio (SNR; from −15 to 15 dB SNR). We defined SNR as the ratio of the average sound pressure of a signal (*P*_signal_) to the average pressure of background noise (*P*_noise_). The SNR_dB_ was the subtraction of *P*_signal_ minus *P*_noise_ in dB SPL. For example, the chorus background (63 dB) mixed with a song of specific pressure (e.g., 73 dB) resulted in an auditory scene of SNR of 10 dB SNR. In addition, each song of varying pressure level had a 0.25–0.27 s snippet of chorus at the beginning and at the end to standardize the onset and offset across stimuli. Each sound stimulus file was about 2.5 s long (1st snippet chorus (0.274 s), song (1.98 s), 2nd chorus snippet (0.25 s). All sound files were high pass filtered at 250 Hz.

**Figure 1.**
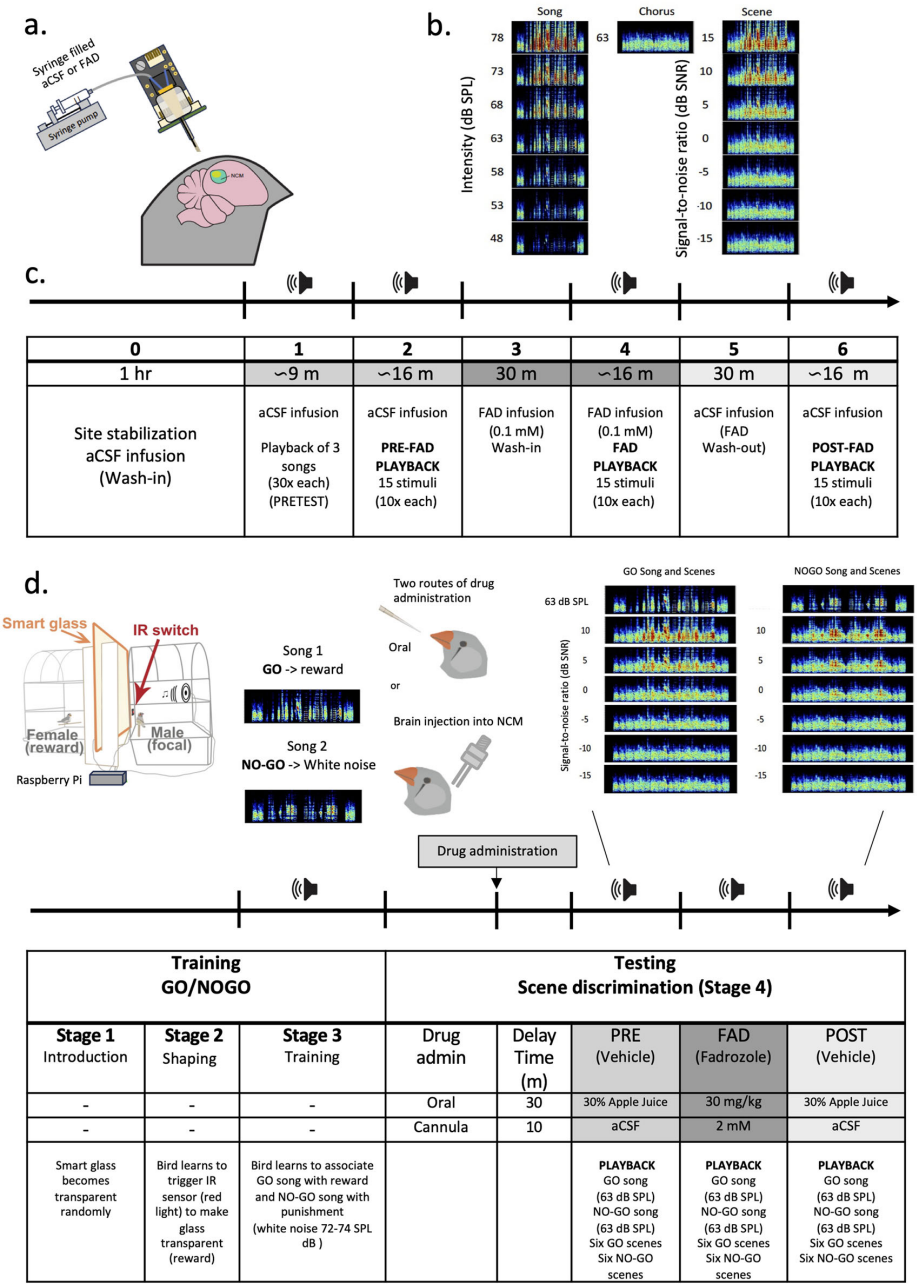
Awake in vivo extracellular recordings in NCM under acute aromatase inhibition. ***a***, RetroDrive (multielectrode drive with 8 tetrodes coupled with a retrodialysis probe). ***b***, Example of a song stimulus set used during playback (song without background amplified to 7 intensity levels 48–78 dB SPL, increment steps of 5 dB) and flanked by 0.25 s chorus snippets, a 63 dB chorus stimulus and 7 auditory scenes of increasing SNR (from −15 to 15 dB SPL) (dB SNR). ***c***, Timeline of the Retrodrive experimental procedure playing back three different song stimulus sets. ***d***, Socially reinforced operant conditioning task used to test discrimination of acoustic scenes under acute aromatase inhibition. The task used a male or female as focal bird and an opposite sex stimulus bird as reward and consisted of four stages. Stage 1: introduction of focal bird to smart glass by becoming transparent at random intervals. Stage 2: shaping of focal bird to peck the infrared (IR) sensor switch. Stage 3: training of focal bird in GO/NOGO associations using conspecific and unfamiliar male songs. Stage 4: drug administration (oral with a micropipette or injection via a bilateral cannula implanted in the NCM region) and testing discrimination of target songs (GO and NOGO) of different intensity levels (48–78 dB SPL) embedded in chorus background noise of constant level (63 dB SPL) resulting in acoustic scenes of different signal-to-noise ratios (SNRs) (aCSF, artificial cerebrospinal fluid; FAD, fadrozole; NCM, caudomedial nidopallium).

In summary, each *song stimulus* set consisted of 15 acoustic stimuli: a 63 dB SPL chorus, and a song played at 7 different dB SPL in quiet and 7 different dB SNR embedded in the chorus background. We had 4 different songs sets to choose from and use following a balanced sampling, in any given experimental trial (Fig. 1).

### Neuronal discrimination of songs in auditory scenes

We used female (*n* = 6) and male (*n* = 4) zebra finches for electrophysiological experiments. All subjects were adults (*x̄* = 323 d post hatch), gonadally intact and with no breeding experience. Two recordings were performed in each bird, once in each hemisphere.

### Surgical procedure

Birds underwent a surgical procedure under anesthesia to attach a head post to enable awake restrained electrophysiological recordings. During the same surgery, bilateral craniotomies were made to gain access to the region above the NCM. Before the surgery, animals were captured from single-sex aviaries and isolated in a clean cage, food deprived for 20 min, given anti-inflammatory (Meloxicam; 0.3 kg/mg) and then placed inside an isoflurane induction chamber (1–2% isoflurane, 1 L/O_2_). After induction, birds were transferred to a stereotaxic apparatus and head-fixed at a 50° angle. After assuring that the bird was stable under anesthesia, a local subcutaneous injection of lidocaine (20 µl, 2% in ethanol) was administered on the site of incision. The bifurcation of the mid-sagittal marked the zero point from which we measured coordinates to reach the NCM region (marked boundaries: rostral 1.2 mm, lateral 1.1 mm) in the left and right hemispheres. We also made a small hole in the upper skull layer above the cerebellum and affixed a 0.6 mm silver ground wire with cyanoacrylate. After, the head post was secured in position using dental cement, we performed bilateral craniotomies by removing both skull layers leaflets and dissecting the dura matter. The craniotomies were then covered with silicone elastomer (Kwik-cast). Birds usually recovered from anesthesia in <5 min and were housed individually but in the company of other birds in adjacent cages for 1–3 d until recording.

### Awake *in vivo* retrodialysis and extracellular recordings in the secondary auditory region NCM

For extracellular recordings, we used custom-built multielectrode drive coupled with a retrodialysis probe (RetroDrives) (Macedo-Lima et al., 2021; Fig. 1*a*). In brief, the RetroDrive consisted of a circular printed circuit board (PCB), soldered to a 36-pin Omnetics connector, a copper magnet wire (stripped at the tips) soldered to the PCB ground and a stainless steel 17 Ga guide tube holding three polyimide tubes (Cole-Palmer). Four tetrodes were then inserted through two of the three polyimide tubes and pinned to the PCB with gold pins (Neuralynx). A reference wire (50 μm polyimide-coated wire; Sandvik) was inserted through the third polyimide tube. Tetrodes were made by twice folding and twisting polyimide-coated 12 µm NiCr tetrode wire (Sandvik) with a tetrode spinner (LabMaker). A microdialysis cannula (CMA8011085; Harvard Apparatus) was glued next to the tubes containing the tetrodes. The cannula protruded 3 mm from the guide tube and tetrodes were cut ∼0.5 mm from the tip of the cannula. The horizontal distance between the cannula and the tetrodes was ∼0.2–0.25 mm. The reference wire was cut at an acute angle at similar length as the tetrodes. Tetrodes were gold-plated to 200–250 kΩ impedance and all wires and pins were covered with liquid electrical tape at the electrode interface board (Gardner Bender). Before inserting the microdialysis probe, recording wires were dipped in 6.25% DiI (Thermo Fisher) in 100% ethanol for later site confirmation of the tube holding the probe and the wires.

For retrodialysis, we used a microdialysis probe (CMA 11 6 kDa 1 mm; Harvard Apparatus) that was perfused with microfiltered artificial cerebrospinal fluid (aCSF) (vehicle; in mM: 199 NaCl, 26.2 NaHCO_3_, 2.5 KCl, 1 NaH_2_PO_4_, 1.3 MgSO_4_, 2.5 CaCl_2_, 11 Glucose, pH 7.4) using a microinjection pump (PHD2000, Harvard Apparatus). FAD (Novartis) was dissolved in aCSF at 0.1 mM and microfiltered. Previous studies have shown that FAD retrodialyzed at this dose into the NCM region causes changes in auditory evoked activity (Remage-Healey et al., 2010). Aliquots 1 ml of the aCSF and FAD were made at the same time and kept frozen until the recording day.

Before the extracellular recordings, we moved the bird inside a sound-attenuating chamber (Eckel Industries). The subject bird was comfortably wrapped with a Kimwipe tissue, placed in a tube holder and head-fixed (50° angle) on an air table (TMC). Throughout the experiment the bird was monitored for any signs of distress and readjusted if any was present. After exposing the craniotomy over one hemisphere, the RetroDrive holding the microdialysis probe inserted into the cannula was lowered to the NCM (∼1.5–2 mm from brain surface; mediocaudally to skull markings). The tetrodes were positioned medially to the probe such that the wires were ∼0.5 mm lateral and 1.2 anterior from the midsagittal sinus (stereotaxic zero).

The recording site was chosen at approximately 1.5–2.0 mm from the brain surface into NCM and when 4 or more tetrodes were active and exhibiting vigorous sound evoked activity. After the site was chosen, we allowed the tissue to stabilized for 1 h after the insertion of the RetroDrive. During this time, aCSF was retrodialysized continuously (retrodialysis speed: 2 μl/min) (Fig. 1*c*). Following the site stabilization period, we performed a pretest playback of 3 songs presented randomly (at 63 dB SLP, without background, 30 repetitions each, interstimulus interval of 5 ± 2 s) to allow the auditory neurons to habituate to these songs (Chew et al., 1995). The same 3 songs used for habituation were used for varying sound level and auditory scene playbacks during treatments (one song set for each treatment). Their presentation order was random. After the pretest period, brain activity was recorded in response to a PRE-playback (a song stimulus set of 15 stimuli played back randomly) under continuing aCSF infusion at the recording site. Then, the aCSF syringe was replaced by one containing FAD (0.1 mM). After a FAD wash-in period of 30 min, brain activity was recorded in response to a different song stimulus set under FAD infusion (FAD playback). The FAD syringe was then replaced by the aCSF syringe which infused vehicle control again for 30 min (FAD wash-out). Finally, brain activity was recorded in response to the final song stimulus set under aCSF infusion (POST-playback) (Fig. 1*c*). At the end of this period, the RetroDrive was slowly retracted, and the craniotomy covered with silicon elastomer (Kwik-cast). The total time of restraint was approximately 4 h. The bird was returned to its cage with food and water *ad libitum*. Recordings from the other hemisphere were performed 1–2 d later by using the same methodology but with a different combination of three song stimuli.

Extracellular voltage was amplified and digitized by a 32 channel head-stage amplifier and evaluation board (RHD2000 series; Intan Technologies). Recordings were sampled at 30 kHz using Open Ephys software. An Arduino Uno delivered TTL pulses to the evaluation board's DAC channel to denote the beginning and end of audio stimuli. A custom-made MATLAB (MathWorks) script controlled the sound playback and TTL pulses from the Arduino and sent a copy of the audio analog signal to the ADC channel of the evaluation board.

**Figure 2.**
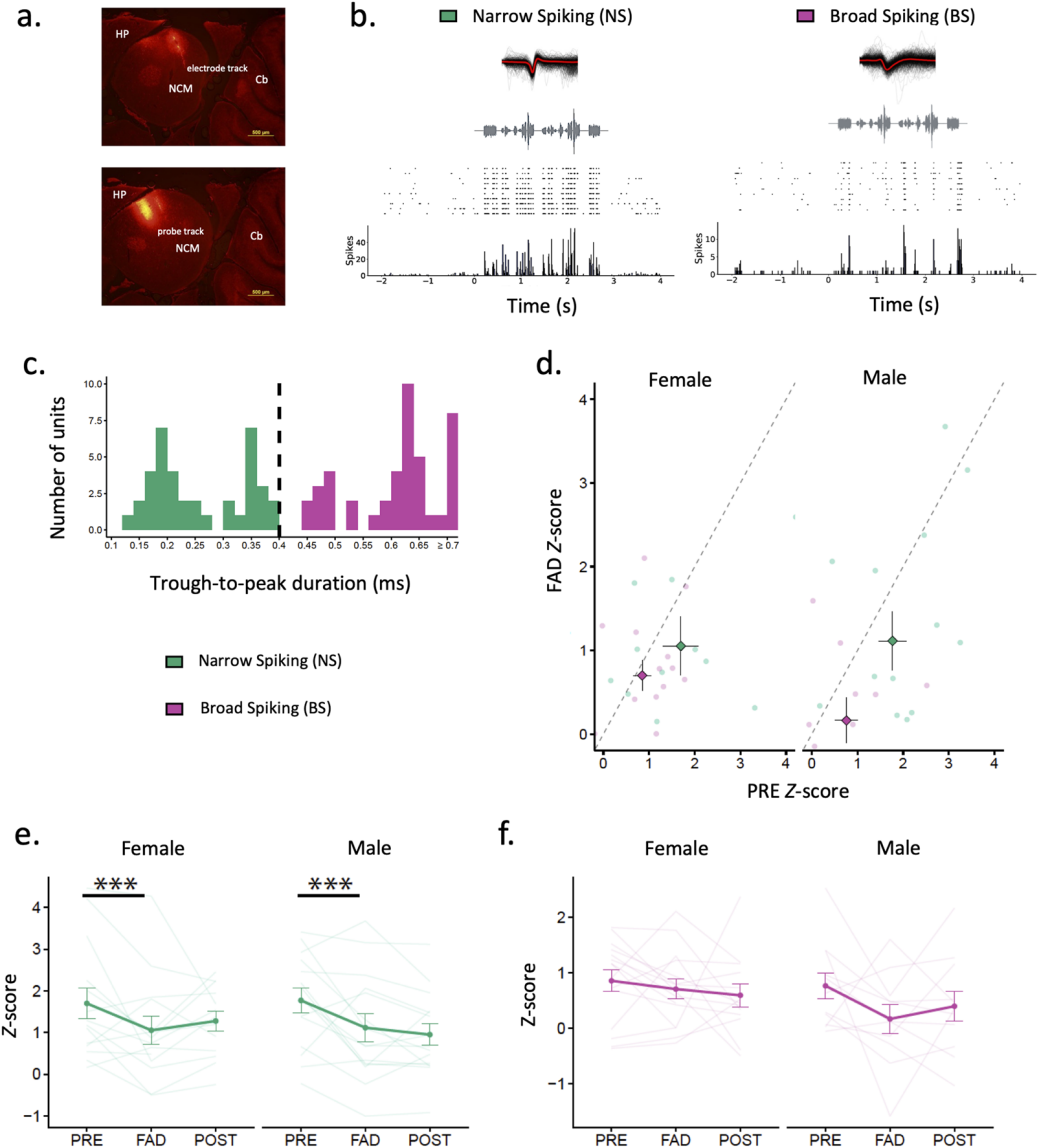
Extracellular recordings in NCM under acute aromatase inhibition. ***a***, Sagittal sections showing recording site location of retrodrive. Retrodrive was dipped in fluorescent dye DiI before penetrating the brain. Track of tetrode wires in the NCM (female 25F31Y) (∼0.2 mm lateral from midline) and track of the microdialysis probe in the NCM (female 25F31Y) (∼0.5 mm lateral from midline). ***b***, Representative PSTHs and waveforms of narrow spiking (NS) (18MB38G19_514) and broad spiking (BS) (17MW159P9_234) cell in response to song B (63 dB SPL) without chorus background. ***c***, Population of NCM neurons sampled classified as NS cells (trough-to-peak durations <0.4 ms) and BS cells (trough-to-peak durations >0.4 ms). ***d***, Mean *z*-scores obtained during PRE and FAD in response to 63 dB songs. Dashed line represents the “line of no change” in *z*-scores between PRE and FAD treatments. Values below the line of no change represent neurons with *z*-scores that tended to decrease after FAD retrodialysis. Values above the dashed line represent neurons that increased after FAD. ***e***, Mean *z*-scores obtained in NS and ***f***, in BS during PRE, FAD and POST and divided by sex. *p < 0.05, **p < 0.01, ***p < 0.001. aCSF, artificial cerebrospinal fluid; Cb, cerebellum; FAD, fadrozole; NCM, caudomedial nidopallium; HP, hippocampus.

On the last day of electrophysiological recordings, immediately following the recording, the bird was anesthetized with isoflurane and quickly decapitated. The brain was extracted and drop fixed in 20% sucrose in 10% formalin solution and stored in 4°C. After fixation, brains were frozen at 20°C for 2 h and then stored at −80°C until sectioning. Brains were sectioned at 40 μm using a cryostat (Leica CM 3050S) and sections were mounted onto gelatinized Fisher SuperFrost slides. Sections were washed in PB for 10–15 m and then coverslipped with Prolong™ Gold Antifade mountant (Invitogen). Sections were visualized under a fluorescent microscope to confirm the location of the probe and wires (Fig. 2*a*).

### Data analysis of *in vivo* electrophysiology recordings

Extracellular recordings obtained from the multielectrode experiment were processed in MATLAB (MathWorks). They were high pass filtered at 300 Hz and common median filtered. Spike single unit sorting was performed by Kilosort (Pachitariu et al., 2016) and results manually curated in Phy (CortexLab). Sound playback timestamps were extracted using a custom-made audio convolution algorithm in MATLAB. Criteria to identify single units in Phy included discarding noisy clusters and identifying possible unit contamination based on spike correlograms. We retained clusters of high SNR, low violation of the refractory period (interspike interval, ISI; within 1 ms) and low overlap with other units according to waveform segregation in the principal component analysis (PCA) space. Final decision on well-isolated units was based on whether the unit fulfilled adequately four conditions: the ISI criteria, high spike amplitude, consistent spike trace over time and sound evoked response consistency judged by visual inspection of peristimulus time histograms (PSTHS) generated using 10 ms time bins (Fig. 2*b*). Finally, sound responsiveness was tested by comparing each neuron's activity before (2 s baseline) versus during playback (all stimuli) during aCSF infusion (PRE) using a Wilcoxon signed-rank test.

After single units were identified, 2,000 waveforms were selected pseudorandomly and their width and symmetry of the action potential (AP) were measured in MATLAB. The AP width was determined by the duration from trough to peak of the average waveform. Several lines of evidence have suggested that neural waveforms in the NCM can be classified into two main and physiologically distinct groups of cells types based on the shape of the waveform of the AP (Schneider and Woolley, 2013; Calabrese and Woolley, 2015; Bottjer et al., 2019; Spool et al., 2021). As in other studies previously published, we observed a nonuniform distribution of waveform durations with an apparent cutoff at 0.4 ms duration of AP width (Krentzel et al., 2018; Macedo-Lima et al., 2021). We then classified single units into two cell types: narrow spiking (NS, AP width <0.4 ms) and broad spiking (BS, AP width >0.4 ms) (Fig. 2*b,c*).

After single-unit sorting, we calculated firing rates spontaneous and stimulus evoked), *z*-scores and spike latency using custom-made Python scripts. Baseline firing rates were calculated using 500 ms preceding each stimulus playback. *Z*-scores were calculated based on the formula $Z=(\mathrm{Mean}\;(S)-\mathrm{Mean}(B))/\sqrt{\mathrm{Var}\;(S)+\mathrm{Var}(B)-2\;(\mathrm{Cov}(S,B)})$, where *S* and *B* are the stimulus and spontaneous firing rates across stimulus trials, respectively.

To assess temporal properties of spiking activity, we measured the latency of a single unit to respond to a stimulus which was the time after the stimulus onset in which the PSTH rose above 3 standard deviations of the average firing during the preceding spontaneous period (0.1 s). The analysis excluded units with latencies higher than 0.4 ms (Ono et al., 2016). To analyze trial by trial consistency in spiking activity (spiking reliability), we ran a customed-pattern classifier (Python) that predicts stimuli discrimination based on spike timing (Krentzel et al., 2018). After 1,000 permutations, the classifier generated a mean spike timing accuracy score in response to each stimulus presented.

To determine neural encoding of songs in auditory scenes, we calculated a song extraction index based on a correlation-based metric of spike timing reliability (*R*corr) obtained from the spike trains of each neuron produced in response to the repeated presentation of the same stimulus (Schneider and Woolley, 2013; Krentzel et al., 2018; Scarpa et al., 2022). A custom algorithm coded in Python correlated pairs of individual spike trains (PSTH binned at 1 ms, smoothed with 20 ms Hanning window). We calculated the correlation between the responses to the auditory scene (song + chorus) and the song without background chorus (*R*corr_song_) at each dB re:100% level (e.g., responses to 0 dB re:100% song correlated with 0 dB re:100% song embedded in chorus) and between the scene (song + chorus) and the chorus background (*R*corr_chorus_). Then, we calculated the song extraction index $\left(R\mathrm{cor}r_{\mathrm{song}}\;\text{–}\;R\mathrm{cor}r_{\mathrm{chorus}})/(R\mathrm{cor}r_{\mathrm{song}}+\;R\mathrm{cor}r_{\mathrm{chorus}}\right)$ for each auditory scene of different SNR. An extraction index below 0 depicted an auditory scene that was more similar to chorus (chorus-like), while an index above 0 depicted a scene whose spike train was more similar to that recorded from the song without background (song-like).

Extraction indices for each scene SNR were used to calculate neurometric thresholds (von Trapp et al., 2016). First, extraction indices values were fit with a sigmoid using the “nlsLM” function from the “mnpack.lm” package for R using the formula $F(x)=\min(EI)+\;\frac{\mathrm{range}(EI)}{1+e^{\frac{x0-x}{b}}}$, where *x* represents the SR values as fractions from 0 dB SNR (i.e., $\mathrm{value}=10^{\frac{\mathrm{dB}}{20}}$), EI represents the extraction index values, and *x*0 and *b* are parameters to be estimated by the fit (starting values: *x*0 = median(*x*) and *b* = 1). Fit validity was verified by (1) the significance (*p* < 0.05) of Pearson's correlations between fit-predicted and actual extraction index values and (2) whether at least one extraction index value was above 0. Neural thresholds were computed from valid fits by determining the point at which the sigmoid curve crossed the extraction index value of 0.

### Behavioral discrimination of songs in auditory scenes

#### Experiment 1: systemic inhibition of aromatase (oral administration)

We used female (*n* = 6) and male (*n* = 8) zebra finches to examine the effects of FAD on behavioral ASA. The behavioral protocol started by pairing opposite sex birds for at least 2 weeks to allow familiarization and bonding in the subject's cage inside the testing chamber. Two weeks of cohousing may promote pair bonding in zebra finches (Zann, 1996). Each testing chamber consisted of an acoustic attenuation chamber (inside dimensions 60.96 × 60.96 × 81.28 cm, Eckel Industries) that isolated the pair of birds from visual and acoustic contact with other birds and humans. The testing chamber was equipped with two cages (focal or subject's and stimulus’) and a visual blockade, a polarized glass sheet (91.4 × 91.4 cm; Smart Tint) positioned between the two cages. The subject's cage had a custom-made switch consisting of an infrared (IR) beam break sensor mounted on a semi-opaque acrylic rectangle that the bird learned to activate to make the opaque magic glass transparent. The acrylic rectangle had a LED red light placed behind it to encourage the bird to peck at the light and break the IR beam (Fig. 1*d*). Birds were only visually but not acoustically isolated. Detailed description of this socially reinforced operant conditioning chamber has been previously described (Macedo-Lima and Remage-Healey, 2020).

After the familiarization period, the stimulus bird was separated from the subject at 2 P.M. and moved into the stimulus’ cage. The next day at 9 A.M., the subject started a 5 h daily training session consisting of five stages (Fig. 1*d*). Stage 1 (Introduction) of the protocol consisted of 2 d in which the glass turned transparent for 6 s in 30–60 s pseudorandom intervals while the activation switch was not operational. During stage 2 (Shaping), the red LED light at the switch turned on at the beginning of the trial. When the IR beam was broken and activated, the polarized glass would turn transparent for 6 s. Between days 1–3, a piece of lab tape baited with egg food supplement was added between the IR sensors. Trials were initiated by the subjects henceforth. The baited tape was not replenished after 2 consecutive days of >70 activations per day. Finally, shaping continued until 2–3 consecutive days of around 100 activations per day.

Stage 3 (GO/NOGO training) consisted of a GO/NOGO paradigm for discrimination of two novel conspecific songs (Fig. 1*d*) played from speakers (SONY) on top of the cage at 63 dB SPL (∼2 s duration). Visual access to the companion bird through the glass was employed as the positive reinforcement while a brief aversive pulse of white noise was used as negative reinforcement. One song was assigned as a GO and another song as the NOGO stimulus to each bird and song assignment was counterbalanced across birds. Birds initiated all trials by triggering the IR beam, after which a song was played from the speaker. After the first trigger, the subject had 2 s window to respond to the stimulus by triggering the sensor again. In a GO trial, visual access to the companion for 6 s (*Hit* response) ensued. In a NOGO trial, the response was registered as a False Alarm (FA) and followed by a white noise pulse (72–74 SPL dB; 2 s) and a 16 s timeout, during which the red LED indicator was remained off. Absence of a response within 2 s after the GO or NOGO playback was considered a Miss and a Rejection respectively. Misses and Rejections were both followed by a brief 6 s timeout period (indicator LED off).

To maximize motivation to engage in the task, initial training started by presenting the GO stimulus in 90% of the trials for 2 d, 75% for the following 2–3 d and finally 50% for the remaining of training. Correctness (%correct) was monitored thereafter until birds performed at >70% correct for at least 2–4 consecutive days. Using this paradigm, the performance typically asymptoted around 70% (Macedo-Lima and Remage-Healey, 2020).

Stage 4 (Scene discrimination under treatment). After birds were trained to recognize target songs, we tested their ability to recognize them when presented mixed with a chorus background at varying SNRs. In this stage, each GO or NOGO stimuli sets were played consisting of the previously used (trained) song (63 dB SPL; unmasked by background), and six auditory scenes of varying SNRs (same song at different levels embedded in chorus; Fig. 1*d*). Stimuli were pseudorandomly preordered in 140-trial blocks (70 GO and 70 NOGO), so that every stimulus type was played 10 times in a block.

To test whether aromatase inhibition affected scene discrimination, “Stage 4” took place daily 30 min following treatments (Fig. 1*d*). We administered three treatments per subject. During pre- and post-treatments, we orally administered 30 μl of 30% Apple juice (vehicle; diluted in distilled water) using a micropipette. Apple juice has been used previously as an appropriate vehicle for FAD (Prior et al., 2014). During FAD treatment, we administered 30 μl of FAD (30 mg/kg). Dose was decided based on previous studies examining the effects of FAD administered peripherally on behavior (Prior et al., 2014; Alward et al., 2016). Birds had to perform at least five 70 trial blocks per treatment before switching to the next treatment. Achieving about five blocks per treatment took between 2 and 8 d.

### Data analysis of behavioral scene discrimination

We analyzed performance in 2 h time windows. Other studies have shown that FAD can act rapidly and transiently (Prior et al., 2014; Alward et al., 2016; Krentzel et al., 2020). Thus, average performance over 5 h would obscure the potential rapid effects of the drug. On average, birds triggered the sensor three to five times per stimulus in 1 h windows and 8–14 trials per stimulus in 2 h windows. Therefore, to assure greater statistical representation per stimulus and temporal resolution of drug action, we divided the data in two 2 h windows.

Performance was examined by the outcome metric of percentage correct or overall performance: $\text{\%}\;\mathrm{Correct}\;=\;(\#\;\mathrm{Hits}\;+\#\;\mathrm{Rejects})/\#\;\mathrm{Trial}s_{\mathrm{all}}*100$. Recent evidence has demonstrated that GO (sum of Hits and Misses) and NOGO trials (sum of Rejects and False Alarm) are learned differently and at a different rates (Gess et al., 2011; Anand and Nealen, 2019; Macedo-Lima and Remage-Healey, 2020). Therefore, we also assessed separately the effects of FAD on Hit Rate, $\mathrm{HR}\;=\;[\#\;\mathrm{Hits}/\#\;\mathrm{Trial}s_{\mathrm{GO}}]*100$, and on Rejection Rate $\mathrm{RR}\;=\;[\#\;\mathrm{Rejects}/\#\;\mathrm{Trial}s_{\mathrm{NOGO}}]*100$.

To determine if subjects exhibited indiscriminate responses, we also examined mean number of trials, mean reaction time, and calculated a measure of response bias c=−0.5*[Z(HH)+Z(RR)] (Macmillan and Creelman, 1990; Macedo-Lima and Remage-Healey, 2020), where Z(HH) and Z(RR) are the inverse cumulative distribution functions at hit and rejection rate (RR) probabilities, respectively. Positive values suggest a tendency to not respond and negative values a tendency to respond indiscriminately.

#### Experiment 2: NCM inhibition of aromatase (brain infusion)

To examine the effects of aromatase inhibition locally in the NCM during behavioral discrimination of auditory scenes, we performed brain injections in bilaterally-cannulated birds. We used female (*n* = 4) and male (*n* = 5) zebra finches that had participated previously in experiment 1. Thus, subjects were experienced on the task and were trained to identify a specific pair of GO and NOGO songs.

### NCM cannulation surgery

Before surgery, each subject underwent the training protocol (Stage 3) for several days to ensure performance was stable and above 70% Correct. Birds underwent cannula implantation surgery following at least 3 consecutive days of performance above criterion. Bilateral cannulas (Plastic One) were custom-made to target the NCM region. They consisted of a pair of guide cannulas (22 Ga and 4 mm in length). The distance between the centers of the barrels was 1.5 mm. The injection (28 Ga) and dummy cannulas (28 Ga) projected 300 μm below the guide cannulas.

For cannula implantation, the subject bird was food deprived for 20 min and received a preemptive dose of Meloxicam (0.3 kg/mg). The bird was then anaesthetized with isoflurane (1–2%) in an induction chamber and transferred to a stereotaxic instrument and maintained anesthetized under isoflurane (0.5–1.5%). The craniotomy over the NCM region was performed following the same steps described above for the head post implantation. The cannula was lowered 1.5 mm ventrally with the stereotaxic apparatus and placed at 1.1 mm anteriorly and 0.7 mm bilaterally, relative to the midsagittal sinus. The head was tilted forward at a 50° angle. The cannula was then secured to the skull and the skin edges sealed with Metabond (C&B) and dental cement (Coltene Whaledent).

The bird was allowed to recover 3 or more weeks in its behavioral cage adjacent to its partner cage with the smart glass turned transparent. After recovery, the Stage 3 of training resumed, and the performance discriminating previously trained GO and NOGO songs was assessed to confirm that auditory performance was not impaired by the surgery. After reaching criterion and maintaining performance for at least 2–3 consecutive days, the bird started “Stage 4” of the protocol to test scene discrimination under control and drug intracerebral treatments (Fig. 1*d*).

After testing, to confirm cannula placement, the bird was euthanized with isoflurane, decapitated, and had its brain extracted using the same methodology described above. Sagittal sections of NCM were stained with thionin and visualized under a light microscope for cannula placement confirmation.

### Brain injections

Treatment rationale was similar to the oral treatments: PRE, followed by FAD and POST. Treatments were only changed after birds completed five 70 trial blocks. For PRE and POST conditions, microfiltered (0.2 micron) aCSF was injected through the cannula. For FAD treatment, FAD (2 mM) was freshly dissolved in aCSF 1 d before the injection. The FAD dose was based on a study that explored the effects of FAD during auditory association learning and discrimination using the same operant task (Macedo-Lima and Remage-Healey, 2020).

Ten minutes before the start of the protocol, the subject bird was captured from its cage in the operant chamber, the bilateral dummy cannula was removed from the guide cannula, and the injection cannula inserted into the guide cannula. Each barrel of the injection cannula was attached to tubing connecting to two 15 μl Hamilton syringes mounted on a syringe pump (Harvard Apparatus PHD2000). A 0.5 μl volume was injected over 1 min for all conditions. Injection cannula was removed between 50 and 60 s after the pump had stopped, the dummy cannula was inserted back into the guide cannula and the bird was returned to its cage. On average, birds were handled for <2 min.

### Statistical analyses

Data obtained from in vivo electrophysiological and behavioral experiments were compared statistically between PRE and FAD treatment periods. POST data was not included in the analyses but plotted in the figures for reference. POST performances were not included in the analysis throughout this study because long term effects of FAD treatments are unclear and could vary across animals. To analyze the electrophysiological data, we explored the effects of treatment on dependent variables in NS and BS neurons using generalized linear mixed models (GLMMs) in R. Due to statistical power limitations, hemisphere effects were not included. The models included Treatment × Sex as fixed effects and Treatment nested under Unit ID and Subject ID as random effects (Table 1).

Behavioral data were analyzed also with mixed models including Treatment × Sex as fixed effects and Treatment nested under Subject ID as a random effect. Data such as % correct, HR, RR and response bias were analyzed with GLMMs in R and number of trials and response or reaction time for GO or NOGO trials were analyzed with linear mixed models (LMMs) in SPSS (Table 1).

We accessed normality of the model residuals by visually inspecting the distribution of studentized residuals obtained from the GLMM (q-q plots) using the “DHARMa” package for R (Hartig, 2021) or tests of normality in SPSS (e.g., Kolmogorov–Smirnov). We reran models after square-root or logarithmic transformation of the data when normality of studentized residuals was not fulfilled or use nonparametric statistics (e.g., Wilcoxon signed-rank test). To transform data containing negative values such as *z*-scores we used [*z*-scores +2 × min|*z*-scores|]. Post hoc pairwise comparisons were evaluated for significant interactions in the model using post hoc Tukey tests (Table 1).

## Results

### NCM coding of auditory scenes

To determine the effects of aromatase inhibition on scene analysis, we recorded in vivo extracellular activity from 81 neurons using 32 channel RetroDrive (microdrive coupled to microdialysis probes; Fig. 1*a*). Of those 81 units, 55 single units were responsive to sound, of which 29 were obtained from 6 female birds and 26 were obtained from 4 male birds. Based on previous studies (Bottjer et al., 2019; Spool et al., 2021), units were classified into two cell types based on their waveform peak-to-peak duration (i.e., action potential width) (Fig. 2*c*). Overall, 47% of the neurons were classified as broad spiking cells (BS) (AP width > 0.4 ms) and 53% were classified as narrow spiking cells (NS) (AP width < 0.4 ms). BS cells included male (*n* = 8 right, *n* = 3 left) and female (*n* = 3 right, *n* = 12 left) units. NS cells also divide in male (*n* = 7 right, *n* = 8 left) and female (*n* = 8 right, *n* = 6 left) units (Fig. 2*c*).

#### Aromatase inhibition decreased normalized evoked responsiveness and timing-based accuracy in narrow spiking (NS) neurons

**Figure 3.**
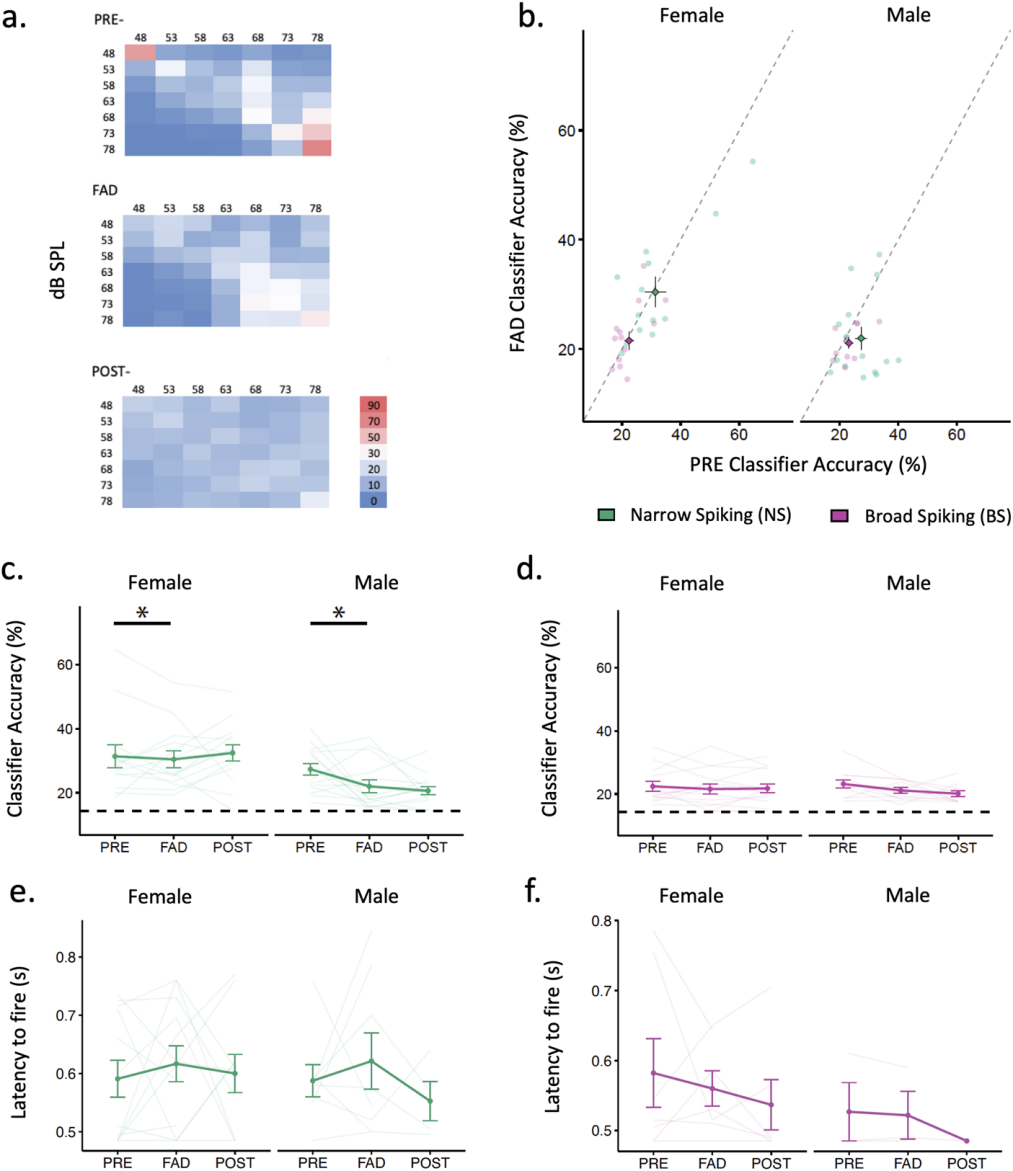
Temporal measures of evoked activity during the retrodialysis of aCSF (PRE playback), fadrozole (FAD playback) and aCSF (POST playback). ***a***, Heat map of the confusion matrix of time-based accuracy of song classification (in response to songs of different dB SPL level without chorus background for a NS neuron (#1209, male 20MLB46W163, right) across treatments. Chance-level decoding accuracy was 14%. ***b***, Mean Classifier Accuracy (%) across levels obtained in Narrow Spiking (NS) and Broad Spiking (BS) neurons during PRE and FAD. Dashed line represents the “line of no change” in accuracy % between PRE and FAD treatments. Values below the dashed line represent neurons with a % that tended to decrease after FAD. ***c***, Classifier Accuracy (%) of NS cells across treatments divided by sex. ***d***, Classifier Accuracy (%) of BS cells across treatments divided by sex. ***e***, Latency to fire (s) of NS units with valid latency divided by sex. ***f***, Latency to fire (s) in BS units with valid latency divided by sex. *p < 0.05, **p < 0.01, ***p < 0.001. aCSF, artificial cerebrospinal fluid; FAD, fadrozole.

The retrodialysis of FAD tended to decrease average normalized responsiveness (*z*-scores) obtained in response to 63 dB song (Fig. 2*d*). The decrease in *z*-scores was statistically significant in both male and female NS neurons (Fig. 2*e*) (treatment *X*^2^_(1)_ = 12.41, *p* = 0.00043). However, a decrease in *z*-scores did not reach significance in BS neurons (treatment *X*^2^_(1)_ = 3.55, *p* = 0.059) (Fig. 2*f*). In addition, we assessed temporal measures of evoked activity such as spiking reliability, and latency of spiking in response to songs at different intensity levels. Spiking reliability was assessed by accuracy scores obtained from a spike timing-based discrimination classifier (% classifier accuracy) previously published (Krentzel et al., 2018; Schroeder and Remage-Healey, 2021; Spool et al., 2021; Fig. 3*a*). We found that FAD tended to decrease classification accuracy in some cells and not in others (Fig. 3*b*). Specifically, FAD decreased classification accuracy of NS neurons in both sexes (treatment *X*^2^_(1)_ = 4.5, *p* = 0.034) (Fig. 3*c*). FAD treatment did not change accuracy in BS neurons (treatment *X*^2^_(1)_ = 1.32, *p* = 0.251) (Fig. 3*d*). In addition, FAD also did not influence response latency in NS neurons (treatment *X*^2^_(1)_ = 0.878, *p* = 0.349; sex *X*^2^_(1)_ = 0.000, *p* = 0.986; treatment × sex *X*^2^_(1)_ = 0.016, *p* = 0.900) or BS neurons (treatment *X*^2^_(1)_ = 0.302, *p* = 0.583; sex *X*^2^_(1)_ = 1.466, *p* = 0.226, treatment × sex *X*^2^_(1)_ = 0.064, *p* = 0.800) (Fig. 3*e,f*).

#### Aromatase inhibition by FAD affected ASA in both cell types in females

**Figure 4.**
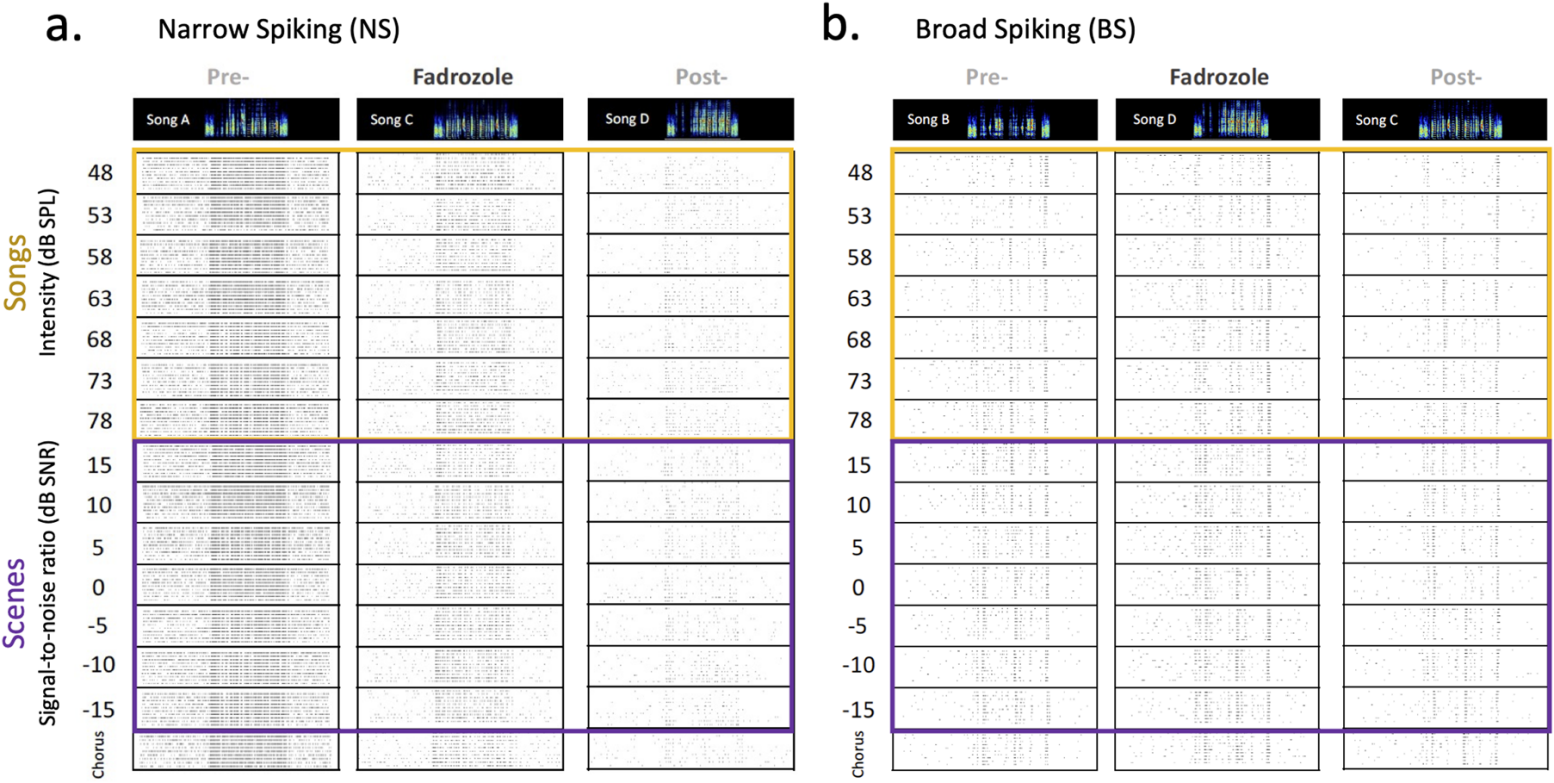
Evoked spike trains obtained from two single NCM neurons in response to songs, scenes and chorus. Songs at different levels (highlighted in yellow), scenes at different signal-to-noise ratios (highlighted in purple) and chorus (63 dB SPL). ***a***, A narrow spiking cell (#207, female 21F15L16L, right hemisphere) during the retrodialysis of artificial cerebrospinal fluid (aCSF) (PRE-FAD playback song A), fadrozole (FAD playback song C) and aCSF (POST-FAD playback song D). ***b***, Broad spiking cell (#234, male 17MW159P9, right) during aCSF (PRE-FAD playback song B), fadrozole (FAD playback song D), and aCSF (POST-FAD playback song C). Top rows show spectrograms of the respective stimulus song of the set at 63 dB SPL and no background.

Spike trains of NS and BS neurons changed progressively with sound intensity of song and SNRs of scenes, as expected, and this measure of ASA was affected by FAD in some neurons (Fig. 4). To examine neuronal song-in-noise discrimination based on temporal correlations obtained between spike trains, we calculated an extraction index (EI) (Schneider and Woolley, 2013) (see Materials and Methods). A positive index value suggests that firing patterns are similar between song (without background) and auditory scene of matching sound levels, suggesting that the firing pattern can discriminate the target song from the background (i.e., song-like spike train). A negative index value suggests that firing patterns are more similar between chorus (background) and scene (i.e., chorus-like spike train) and that there is no discrimination of song from the scene. Schneider and Woolley (2013) reported that in male zebra finches, NCM neuronal discrimination of song from scenes increases as SNRs of scenes increase in a psychometric function. On average, NS neurons discriminate song from scenes 5 dB SNR or higher, whereas BS neurons discriminate song from scene 0 dB SNR or higher (BS have a lower threshold than NS neurons) (Schneider and Woolley, 2013).

**Figure 5.**
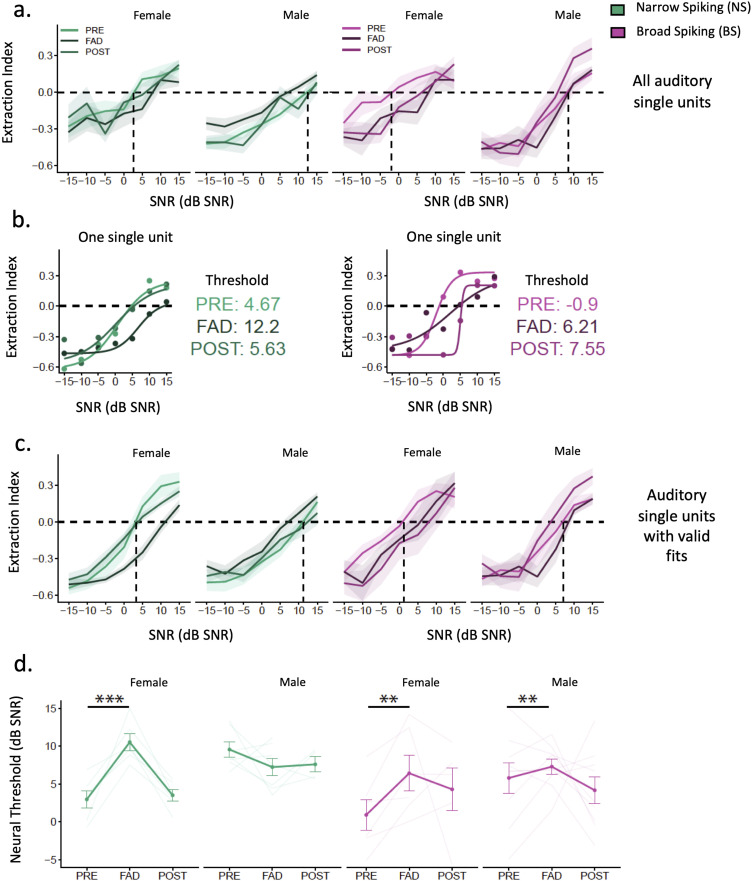
Extraction Index (EI) values obtained from NCM neurons in response to scenes of −15 to 15 dB SNR. Index values above 0 represent scenes that evoked a song-like spike train and index values less than 0 represent scenes that evoked a chorus-like spike train. ***a***, EI for Narrow Spiking (NS) (*n*_female _= 14, *n*_male _= 15) and Broad Spiking (BS) (*n*_female _= 15, *n*_male _= 11) neurons during aCSF (PRE and POST) or FAD treatment. ***b***, Two cells with a valid sigmoid fit during PRE for which a threshold was calculated to determine the signal-to-noise ratio (SNR) at which the sigmoid crosses the 0 extraction index. Higher threshold denotes a poorer ability to extract song from chorus. ***c***, EI for single units with a valid sigmoid fit. ***d***, Mean threshold to extract song between PRE and FAD increased in both female cell types and only in male BS neurons. *p < 0.05, **p < 0.01, ***p < 0.001. aCSF, artificial cerebrospinal fluid; FAD, fadrozole.

Our psychometric curves under aCSF showed that female NS neurons extracted song from scenes (EI values >1) between 0 and 5 dB SNR and BS extracted song from scenes between −5 and 0 dB SNR, confirming a background-invariant feature of BS neurons in females that was previously reported in males by Schneider and Woolley (2013). In our sample, however, male neurons extracted song from scenes around 10 dB SNR (higher than expected) (Fig. 5*a*). Moreover, under FAD psychometric curves shifted to the right in both cell types and sexes, except for male NS neurons (Fig. 5*a*). For cells that exhibited a sigmoid fit, we calculated a threshold value that indicates the minimum SNR at which the sigmoid curve crosses the 0 extraction index value (Fig. 5*b,c*). Therefore, it indicates the mean minimum SNR at which the neuron can discriminate a song within the background chorus. We found that NS cells exhibited high thresholds to code song from background during FAD retrodialysis in females, while in males thresholds between PRE and FAD were not statistically different (treatment × sex, *X*^2^_(1)_ = 23.4, *p* < 0.001; post hoc *t* test female PRE-FAD vs FAD, *t*_(18)_ = 5.19, *p* = 0.001, male PRE-FAD vs FAD: *t*_(18)_ = 1.5, *p* = 0.15) (Fig. 5*d*). Males showed higher thresholds during aCSF retrodialysis than females since the beginning suggesting that male neurons needed better SNRs to extract song from scenes (post hoc *t* test PRE female vs male, *t*_(18)_ = 4.86, *p* = 0.001; FAD female vs male, *t*_(18)_ = 1.97, *p* = 0.064). In BS cells, we found a significant main effect of treatment suggesting that FAD increased the threshold across sexes (treatment *X*^2^_(1)_ = 9.47, *p* = 0.0021) and a trending treatment × sex interaction (treatment × sex *X*^2^_(1)_ = 3.8, *p* = 0.0511) (Fig. 5*d*).

In sum, FAD decreased song extraction index values (and increased neural thresholds) in both cell types, particularly in females. Performance of male neurons was on average low under the control condition; making the effect of FAD difficult to resolve. However, blocking neuroestrogen synthesis did affect the processing of songs embedded in auditory scenes in female neurons.

### Behavioral discrimination of songs in auditory scenes

#### Acute systemic aromatase inhibition reduces RRs of NOGO scenes in females

In total, 14 zebra finches (six female and eight male) learned to correctly discriminate and recognize two songs (a “GO” and a “NOGO” song) in our operant conditioning paradigm. The proportion of birds that did not complete the experiment was either not motivated throughout the task, could not learn how to activate the sensor, or did not reach learning criterion during training. These proportions were not different by sex (*X*^2^_(1)_ = 0.06, *p* = 0.8). On average, male subjects spent significantly more days in Stage 2 (shaping) than females (males *x̄* = 9.2 ± 1.71, females *x̄* = 4.17 ± 0.7 d, *t*_(5) _= 2.71, *p* = 0.042), but both sexes spent similar average number of days training to reach criterion in Stage 3 (GO/NOGO training) (males *x̄* = 27.8 ± 3.4, females *x̄* = 25.7 ± 7.3 d, *t*_(7) _= 0.26, *p* = 0.8). After subjects reached 70% correct performance during training for more than 2–3 consecutive days, we tested the effects of an oral administration of 30 µl of either vehicle (30% apple juice) or FAD (30 mg/kg) on the ability to discriminate the GO song embedded in background chorus (an auditory scene) at different SNRs.

**Figure 6.**
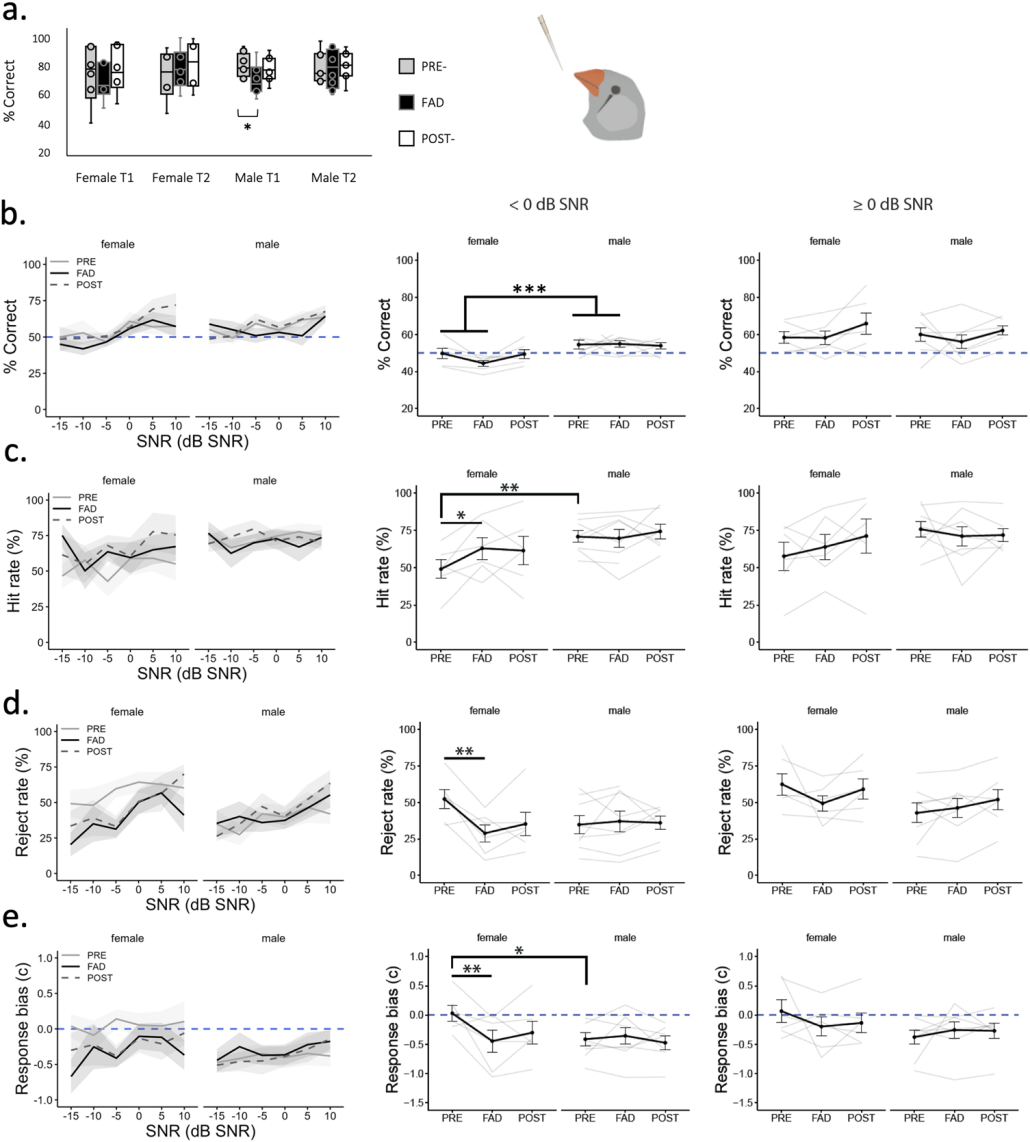
Behavioral discrimination of auditory scenes after a single daily oral administration of vehicle control (PRE), fadrozole (FAD) and control (POST). ***a***, Female and male behavioral performance (% correct) at discriminating GO song at 63 dB SPL (no chorus background) during 2 h time window 1 (T1) and time window 2 (T2). ***b–e***, Behavioral performance across scenes and divided by scenes of low signal-to-noise ratio (<0 dB SNR) and high SNR (≥0 dB SNR), ***b***, % Correct ((#Hits+#Rejects)/#Trials_All_ *100)), ***c***, Hit rate ((#Hits)/#Trials_GO_ *100) and ***d***, Rejection rate ((#Rejects)/#Trials_NOGO_ *100), ***e***, Mean response bias (positive values indicate bias towards not responding and negative values indicate bias towards responding) *p < 0.05, **p < 0.01, ***p < 0.001.

After the oral administration of vehicle, male and female birds correctly discriminated the 63 dB SPL GO song without background on average (females 70.8 ± 4.5% correct and males 73.6 ± 3.9% correct) (Fig. 6*a*). During the first 2 h (time window T1) of the test, performance (% correct) during FAD was significantly lower in males (Wilcoxon signed-rank test: *z* = −1.96, *p* = 0.05, *d* = 0.69) and performance was lower but not significantly so in females (Wilcoxon signed-rank test, *z* = −0.524, *p* = 0.6). Based on this and previous work demonstrating the rapid circulation and elimination of FAD in the body after a peripheral administration (Prior et al., 2014; Alward et al., 2016), we focused our analyses on time window 1 (first 2 h of testing after the drug administration).

Song discrimination embedded in scenes, measured by % correct, increased from −15 to 10 dB SNR scenes, but was not affected by FAD (Fig. 6*b*). Based on previous work, it is expected that birds achieve higher performance in response to auditory scenes of higher SNRs (≥0 dB SNR) than to scenes of lower SNRs (<0 dB SNR) (more challenging). When we assessed mean performance in response to scenes of high and low SNR, males performed better (% correct) than females discriminating song in scenes of low SNR (<0 dB SNR) regardless of treatment (sex, *X*^2^_(1)_ = 13.5, *p* = 0.0002). In response to GO trials by examining hit rate (HR) in response to low SNR scenes (<0 dB SNR), there was a greater HR in females than in males during FAD (treatment × sex, *X*^2^_(1) _= 4.81, *p* = 0.028; post hoc *t* test female PRE-FAD vs FAD, *t*_(21)_ = 2.57, *p* = 0.017; male PRE- vs FAD, *t*_(21)_ = 0.38, *p* = 0.71). Males scored higher HR than females before FAD (post hoc *t* test PRE female vs male, *t*_(21)_ = 2.97, *p* = 0.073; FAD female vs male, *t*_(21)_ = 0.78, *p* = 0.44) (Fig. 6*c*). Thus, FAD improved HR of GO trials in response to low SNR scenes in females.

In contrast, oral treatment of FAD impaired performance in NOGO trials by decreasing RRs (or increasing the proportion of false alarms) in females but not in males (Fig. 6*d*). This decrease in RR is observed when female birds needed to discriminate song from scenes of low SNR (<0 dB SNR) (treatment × sex, *X*^2^_(1)_ = 9.35, *p* = 0.002; post hoc *t* test female PRE-FAD vs FAD, *t*_(21)_ = 3.82, *p* = 0.001; male PRE-FAD vs FAD, *t*_(21)_ = 0.195, *p* = 0.85) (Fig. 6*d*). When discriminating song from scenes of high SNR (≥0 dB SNR), birds showed a not statistically significant interaction (treatment × sex, *X*^2^_(1)_ = 3.18, *p* = 0.0745).

An increase in HRs and decrease in RRs (and thus an increase of false alarms) under FAD could have been the result of indiscriminate responding (response bias). To examine this possibility, we calculated response bias in which positive values indicate bias towards not responding and negative values indicate bias towards responding (see Materials and Methods). Across SNRs, FAD tended to affect response bias in females (Fig. 6*e*). A significant change in bias in females was evident in response to scenes of low SNR (Fig. 6*e*) (treatment × sex, *X*^2^_(1)_ = 8.10, *p* = 0.004; post hoc *t* test female PRE-FAD vs FAD, *t*_(21)_ = 3.35, *p* = 0.003; male PRE-FAD vs FAD, *t*_(21)_ = 0.47, *p* = 0.64). Thus, under FAD females were more biased toward responding (more hits and more false alarms) when exposed to difficult auditory scenes (<0 dB SNR). FAD could have increased bias in females by increasing the number of trials or activations and by reducing the response time. However, female and male birds activated the sensor at a similar frequency between treatments (mean number of trials, Wilcoxon signed-rank test, females, *z* = 1.15, *p* = 0.249; males, *z* = 0.98, *p* = 0.327). Males exhibited faster response times than females in response to NOGO scenes (sex, *F*_(1, 22.7) _= 4.76, *p* = 0.04). But, response time of either sex was not significantly affected by FAD treatment when exposed to scenes with the GO (treatment × sex, *F*_(1,20.17) _= 1.218, *p* = 0.281) or NOGO (treatment × sex, *F*_(1,22.7) _= 0.722, *p* = 0.404) song embedded.

In summary, our results suggest that FAD improved ASA of GO trials and impaired ASA of NOGO trials of low SNR scenes by increasing response bias in female zebra finches.

### Acute bilateral NCM aromatase inhibition increases performance in scene discrimination in females

**Figure 7.**
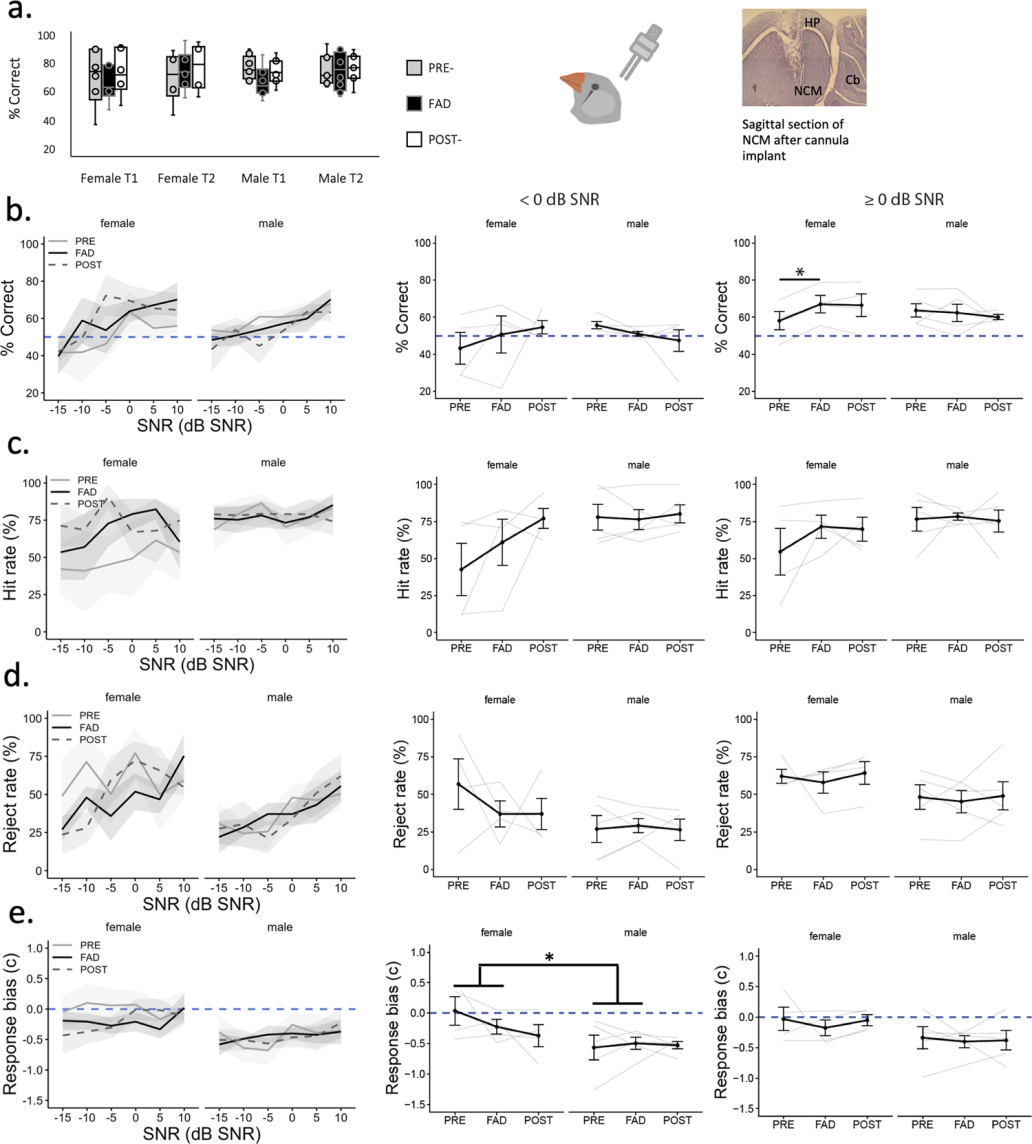
Behavioral discrimination of auditory scenes after a single daily bilateral brain injection of control (PRE), fadrozole (FAD) and control (POST) into the NCM region. ***a***, Behavioral performance (% correct) at discriminating GO song at 63 dB SPL (no chorus background) during 2 h time window 1 (T1) and window 2 (T2). ***b–e***, Behavioral performance across scenes and divided by scenes of low SNR (<0 dB SNR) and high SNR (≥0 dB SNR) ***b***, % Correct ((#Hits+#Rejects)/#Trials_All_ *100)), ***c***, Hit rate ((#Hits)/#Trials_GO_ *100), ***d***, Rejection rate ((#Rejects)/#Trials_NOGO_ *100), ***e***, Mean response bias (positive values indicate bias towards not responding and negative values indicate bias towards responding). SNR, signal-to-noise ratio; Cb, cerebellum; FAD, fadrozole; NCM, caudomedial nidopallium; HP, hippocampus. *p < 0.05, **p < 0.01, ***p < 0.001.

Nine birds (4 females and 5 males) previously used in the experiment with oral administrations were bilaterally cannulated in the secondary auditory cortical area (NCM). After the brain injections of aCSF or FAD, performance (% correct) in response to a song 63 dB without background was not significantly different between treatments during time window #1 in males (Wilcoxon signed-rank test, *z* = −1.48, *p* = 0.138) or in females (Wilcoxon signed-rank test, *z* = 1.46, *p* = 0.144). Male and female birds correctly discriminated the 63 dB SPL GO song without background despite treatment (female 77.1 ± 4.75% correct, male 75.4 ± 2.45% correct) (Fig. 7*a*).

Overall, birds improved scene discrimination (% correct) from −15 to 10 dB SNR scenes regardless of treatment infused directly into NCM (Fig. 7*b*). FAD improved the discrimination of scenes compared to vehicle in females, and not in males, but only in response to scenes of high SNR and when the challenge of the task is not expected to be great (treatment × sex, *X*^2^_(1) _= 6.87, *p* = 0.009; post hoc *t* test female PRE-FAD vs FAD: *t*_(12)_ = 3.08, *p* = 0.009, male PRE-FAD vs FAD: *t*_(12)_ = 0.49, *p* = 0.63) (Fig. 7*b*). When examining HR of the GO trials and RR of the NOGO trials separately, a change in HR after FAD was not significant when testing mean HR between PRE and FAD in low (Treatment, *X*^2^_(1)_ = 1.26, *p* = 0.26) or in high SNR scenes (Treatment, *X*^2^_(1)_ = 1.90, *p* = 0.17) (Fig. 7*c*). Similarly, RR was not significantly affected by FAD either in low (Treatment, *X*^2^_(1)_ = 0.88, *p* = 0.346) or in high SNR scenes (Treatment, *X*^2^_(1)_ = 0.44, *p* = 0.5) (Fig. 7*d*).

After FAD infusions, response bias was mainly unaffected (Fig. 7*e*). In response to scenes of low SNR, there was a significant main effect of sex in which females were less biased than males, but this was regardless of treatment (Sex, *X*^2^_(1)_ = 6.28, *p* = 0.012). We found no evidence that FAD increased the average number of trials or activations performed by the birds of either sex during testing (treatment × sex: *F*_(1,11.6) _= 0.11, *p* = 0.746). FAD did not affect response times across SNRs to GO (treatment × sex, *F*_(1,11.3) _= 0.248, *p* = 0.628) or NOGO scenes (treatment × sex, *F*_(1,12) _= 0.01, *p* = 0.923) in either sex.

The results obtained with the oral treatment did not match the results obtained with the NCM injections suggesting that the effects observed after oral FAD treatment were not localized to the NCM specifically. The lack of a significant detrimental effect, and a rather positive effect, of FAD in females (Figs. 6*c*, 7*b*) could be explained by rapid secondary effects of the drug. Previous studies have reported testosterone (T) levels rise locally after the administration of oral or brain FAD (Remage-Healey et al., 2008; Prior et al., 2014; de Bournonville et al., 2020).

## Discussion

To our knowledge, this study is first in examining the role of neuromodulators in ASA in male and female songbirds. Recording from the secondary auditory pallial cortex (NCM), we found that the aromatase inhibitor FAD application impaired the capacity of NS and BS neurons to extract song from auditory scenes, particularly in female subjects. NS neurons also showed a decrease in auditory normalized song responsiveness and song timing-based classification. Behaviorally, systemic aromatase inhibition by FAD increased bias to respond indiscriminately in females, and not in males. Consequently, we observed an increased proportion of hits and false alarms during scene discrimination. Aromatase inhibition directly within the NCM modestly improved performance in females, but this difference may have been driven by a lower performance exhibited at PRE-FAD or secondary effects of the drug. Thus, the impaired performance of NCM neurons in scene extraction did not directly lead to a behavioral disruption in ASA, indicating a resilience, redundancy, and/or compensatory responses. Moreover, these results highlight the potential role of other aromatase-rich brain regions outside the auditory pathway involved in ASA.

### Effects of aromatase inhibition on NCM neural responses to songs in quiet background

We found that FAD decreased auditory responses in putatively inhibitory NS neurons. In songbirds, the NCM is characterized by neurons of heterogenous neurochemical identity (Ikeda et al., 2017; Pagliaro et al., 2020) and a wide range of response properties (Bottjer et al., 2019). About 65% of neurons in the ventral NCM express aromatase, a small number of them co-express the inhibitory marker parvalbumin but the rest are likely to be represented by a diverse composition of neuronal subtypes (Ikeda et al., 2017). In previous reports, FAD decreased response strength to acoustic stimuli in females (Remage-Healey et al., 2012), and affected bursting activity in response to conspecific song in males (Remage-Healey et al., 2010). These studies, however, examined the effects on song responsiveness without dividing the analysis into cell types as we do in the present analysis. NCM neurons can be identified into NS and BS neurons based on their action potential widths (Schneider and Woolley, 2013; Calabrese and Woolley, 2015; Bottjer et al., 2019; Spool et al., 2021). Genetically identified cell types confirmed that GAD1-expressing neurons had narrow AP widths while CaMKlla neurons had broad AP widths (Spool et al., 2021). GAD1 and CaMKIIa are genes that code for enzymes involved in the production of GABA and calcium signaling respectively. Thus, our NS neurons were likely inhibitory interneurons and BS neurons excitatory projection neurons. Inhibitory interneurons in the NCM also colocalize G-protein-coupled estrogen receptor 1 (GPER1), a membrane receptor involved in mediating rapid effects of neuroestrogens, and blocking these receptors also reduced responsiveness in response to song in male zebra finches in vivo (Krentzel et al., 2018). Thus, in our study, FAD may have reduced available estrogenic binding to these membrane receptors in inhibitory interneurons and reduced their activity in response to song playback. Based on a cortical excitatory–inhibitory balance (Froemke, 2015), one would expect FAD to also impact song responsiveness in excitatory BS neurons when inhibition is disrupted by FAD. But we did not observe changes in responsiveness in BS neurons (Fig. 2*f*). Therefore, although a dynamic balance between excitation and inhibition is certainly necessary to process complex auditory input, it is recognized that those dynamics are difficult to elucidate with extracellular recordings alone (Froemke, 2015).

Blocking estrogen synthesis was also expected to affect other firing properties such as temporal patterning and firing precision, especially if inhibitory neurotransmission is affected. Reliability of NCM spiking in response to song was modulated by blocking GABAergic transmission in starlings (Perks and Gentner, 2015). Recent in vitro work demonstrated that estradiol can rapidly modulate firing precision in NCM neurons with low membrane input resistance (Scarpa et al., 2022). Our pattern classifier results assessing how the auditory response predicted song discrimination based on spike timing showed that FAD reduced classification accuracy in inhibitory NS neurons, and not in BS neurons (Fig. 3*c*). Thus, blocking the enzymatic activity of aromatase affected inhibitory neuronal activity that could impair timing-based auditory perception of auditory scenes (see below).

### Effects of aromatase inhibition on neural and behavioral ASA in the NCM

To assess ASA, we examined the effect of FAD on song extraction indices calculated based on the spiking activity obtained in response to auditory scenes. We found that both NCM NS and BS neurons performed better during PRE- than during FAD retrodialysis (Fig. 5). Processing ASA or complex auditory stimuli requires temporal specificity, reliability, and integration of the spiking activity (Moore et al., 2013; Schneider and Woolley, 2013; Perks and Gentner, 2015). The inhibition of neuroestrogen synthesis could have affected the profiles of excitation and inhibition as well as the temporal precision of the evoked activity enough to impair ASA in both cell types. We found evidence that FAD affected responsiveness and timing-based accuracy of inhibitory neurons (NS). Timing-based accuracy relies on temporal correlation of firing and this temporal disruption could have influenced the ability of NS neurons to extract song from scenes. On the other hand, BS neurons are considered to have greater ability to extract songs from complex scenes due to their sparse coding (Schneider and Woolley, 2013) and longer integration times compared to NS neurons (Moore et al., 2013). The sparse spiking of BS neurons has been shown to be modulated by a GABAergic mechanism of forward suppression (Schneider and Woolley, 2013; Spool et al., 2021). FAD may have affected the inhibitory input to excitatory BS neurons, disrupting their ability to extract song, without an effect on firing rate *z*-scores, accuracy or latency. Alternatively, the lack of estradiol may have affected BS neurons directly rather than via NS neurons in a way that decreased ASA but not firing rate *z*-scores or accuracy. A similar proportion of inhibitory GAD1 and CaMKIIa excitatory neurons have been observed to co-express aromatase in the NCM (Spool et al., 2022), but the functional neurophysiology outcomes for the network needs more study.

We found a clear effect of FAD on song extraction from scenes in females. Male-female comparisons in the ventral and dorsal regions of the NCM have shown no sex differences in the percentage of aromatase expressing cells or of aromatase positive cells that co-express the inhibitory marker parvalbumin (PV) (Ikeda et al., 2017). Nonetheless, females have less aromatase expression in synaptic terminals and fewer somato-somatic clusters of aromatase cells in the NCM compared to males (Saldanha et al., 2000a; Rohmann et al., 2007; Ikeda et al., 2017). Thus, sex differences in aromatase distribution would have predicted a larger effect of FAD in males than in females. In addition, dialysate estradiol levels in the NCM are similar at baseline and equally rise in response to song playback in adult males and females (Remage-Healey et al., 2012). A possible explanation is that males have compensatory mechanisms that can prevent the detrimental effects of blocking the production of neuroestrogens during ASA. As mentioned before, the NCM is a complex associate region that is likely to be functionally modulated by a diverse array of neurochemical and interneurons subtypes (Spool et al., 2022). Alternatively, stronger effects of FAD were not observed in males because our sample of male neurons simply performed poorly at extracting song from scenes regardless of treatment. Thus, detrimental, and clearer effects of aromatase inhibition on male ASA could still be a possibility in future experiments.

It should be also noted, however, that the sex and cell type comparisons constrain the available sample sizes per group. The approach using electrophysiological recordings is often challenged by extracting viable voltage signals and stability during experiments in awake subjects. Moreover, this study of ASA required the presentation of 15 acoustic stimuli of varying SNRs which extended the time of acoustic exposure per treatment. Consequently, the number of viable single units that were stable throughout all stages of the procedure (e.g., stabilization, drug infusion and drug washout) was restricted. Despite these limitations, this study lays the groundwork for future work on neuromodulation of ASA in particular with a fulsome examination of potential sex differences in this and other brain regions.

Finally, acute injections of FAD before ASA using an operant conditioning paradigm demonstrated that aromatase inhibition directly into the NCM did not compromise behavioral ASA in males. Previous work found that NCM injections of FAD did not affect performance in discriminating previously learned tones, only during the association learning of new sounds and contingencies (Macedo-Lima and Remage-Healey, 2020). In the current study, blocking aromatase only locally in NCM impaired ASA at the level of the neurons (electrophysiology) but it did not impair ASA behaviorally. This suggests that NCM is not the only brain region involved in ASA (see below). Alternatively, the buildup of testosterone (T) when aromatase is inhibited could have affected the efficacy of FAD treatment. Retrodialysis of FAD into NCM increases T levels in the NCM of males (Remage-Healey et al., 2008) and in the left hemisphere of females (de Bournonville et al., 2020). Although nonsteroidal type II aromatase inhibitors such as FAD are effective at inactivating the aromatase enzyme, they are usually reversible and have a relatively short-half life (Buzdar, 2006; Miller, 2006). FAD effectiveness depends upon the continued presence of the inhibitor (Buzdar, 2006; Miller, 2006) which was unlikely during this behavioral/cannulation experiment. Thus, the lack of detrimental effects of FAD during behavioral ASA could have been caused by (1) the rapid clearance of the drug and/or by (2) compensating effects of androgens during song processing. Our electrophysiology experiment circumvented this by the continuous retrodialysis of the drug, but the cannulation experiment allowed only a single injection per day. In females, a single FAD injection caused an unexpected improvement in discriminating song from scenes of high SNR during FAD compared to PRE. However, the difference may be attributed to poor initial performance or to stress sensitivity of females during control intracranial injections which took place before FAD injections (Fig. 7).

### Sex-specific effects of systemic aromatase inhibition on behavioral ASA

We successfully trained and tested male and female birds and found that both sexes can perform ASA under baseline conditions. Most previous studies tested only male subjects (Vignal et al., 2004; Narayan et al., 2007; Schneider and Woolley, 2013) or were underpowered to assess sex differences in zebra fiches (e.g., one to four females, two to four males) (Benney and Braaten, 2000; Lohr et al., 2003; Dent et al., 2009). Although the current study also tested a limited number of individuals, it included more birds per sex (six females, eight males), allowing sex to be included as an independent variable in the analysis. We found that song discrimination decreased with SNR between the target song and the background chorus noise as reported in previous behavioral studies of ASA using operant conditioning (Narayan et al., 2007; Dent et al., 2009; Schneider and Woolley, 2013). Under systemic treatment with FAD, we found that overall performance at discriminating song in auditory scenes (% correct) was not affected across sexes (Fig. 6*b*). Therefore, global inhibition of aromatization did not to affect auditory discrimination per se, as seen in a previous study using the same operant design to discriminate tones in male zebra finches (Macedo-Lima and Remage-Healey, 2020).

Despite no effects detected in ASA (% correct), under acute systemic treatment with FAD, females experienced a transient impairment at responding correctly to NOGO songs. Specifically, females were more likely to incorrectly respond when hearing auditory scenes containing the NOGO song during the first 2 h window after the drug administration. GO/NOGO discrimination requires the bird to learn two responses: produce a behavioral response when detecting the GO stimulus and suppress that same behavior when detecting the NOGO stimulus, and the two responses are learned independently (Anand and Nealen, 2019). Therefore, it is possible that the GO and NOGO responses are also regulated differently by steroid sensitive brain regions in males and females. FAD treatment could have caused female subjects to behave more impulsively since it increased liberal response bias, without increasing number of activations overall (Fig. 6*e*). A low number of correct rejections (higher number of false alarms) after FAD treatment suggests an inability to suppress a voluntary motor learned response (Anand and Nealen, 2019). Therefore, a systemic inhibition of aromatase may have caused perceptual impairment during behavioral ASA.

Systemic drug manipulations may be difficult to interpret but open numerous paths of unexplored investigation. Zebra finches have a rich expression of aromatase positive cells in the NCM but also in preoptic area (POA), hippocampus, HVC shelf, and the NCL (caudolateral nidopallium) (Saldanha et al., 2000a; Ikeda et al., 2017; Krentzel et al., 2020). Female brains have more aromatase expression in the posterior region of the HVC shelf (Krentzel et al., 2020). Brain activation based on expression of immediate early genes (EGR-1 activation), the HVC shelf shows song-evoked responses (Krentzel et al., 2020) and it is believed to convey auditory information to the motor pathway involved in song production (Vates et al., 1996). In addition, the HVC could be considered a songbird specialization of the dorsal NCL (Farries, 2001). The dorsal region of the NCL is also sound-evoked in males and believed to be involved in auditory-motor integration (Bottjer et al., 2010). The NCL is engaged during GO and NOGO tasks involving visual and auditory stimuli (Kalt and Diekamp, 1999), exhibits a rich neurochemical profile (Güntürkün, 2005; Herold et al., 2011), and is considered to have a major role in executive function and cognition (Güntürkün, 2005; Rose and Colombo, 2005). Sex differences in aromatase expression in the NCL have not been formally examined, but the HVC shelf and the NCL are of special interest by being rich in aromatase expression (Ikeda et al., 2017), potentially sexually dimorphic and involved in high-order processing. The systemic treatment with FAD in our experiments likely blocked aromatization in these regions and could have caused the observed increased in false alarms, and response bias in females, and thus, an inability to suppress a behavioral response.

Why did systemic FAD exposure not affect ASA in males? FAD administered systemically or locally in the brain is known to effectively decrease aromatase activity (Wade et al., 1994; Prior et al., 2014), but such reduction is transient (<4 h) (Alward et al., 2016). Perhaps, the clearing of the drug occurred faster in males and the time at which males activated the sensor did not coincide with FAD's peak action. Our behavioral experiments have the caveat that trials are self-initiated. Secondly, aromatase inhibitors can upregulate testosterone synthesis in human and nonhuman mammals and differences in circulating androgens can in turn affect the maintenance of local estrogen levels in extragonadal sites (Ellinwood et al., 1984; Kochak et al., 1990; Fernández-Vargas, 2017). Chronic systemic administration of FAD increased plasma T levels in intact and castrated male zebra finches and other songbirds (Schlinger et al., 1999; Soma et al., 1999; Moore et al., 2004; Prior et al., 2014). Acute oral administration of FAD also increased plasma T levels in male and female gonadally intact zebra finches (Prior et al., 2014). Therefore, in our experiments it is possible that oral FAD activated the hypothalamic-pituitary gonadal (HPG) axis by disrupting the negative feedback on luteinizing hormone and stimulating the gonads to produce T. An intramuscular injection of T can also increase forebrain levels of T (Remage-Healey et al., 2008), which may have lessened the effectiveness of FAD in reaching absolute estrogen suppression in our experiment. Aromatase expressing areas in the NCM and the HP also express androgen receptors (Metzdorf et al., 1999). The concomitant T elevation plus initial higher baseline levels of circulating T in males compared to females (Agate et al., 2003) could explain why we did not observe decreased performance in males. Therefore, a combination of a potential fast clearing of the drug with androgenic secondary effects could explain the nonsignificant results found in behavioral performance in males. Further exploration using specific androgenic and estrogenic manipulations directly could discriminate among these possibilities.

## Conclusions and broad implications

This work highlights the neurochemical complexity of the auditory network in the songbird NCM and supports the accumulated evidence that neuroestrogens have a significant role in the auditory encoding of behaviorally relevant signals. Improved methods yielding larger data outputs and a combination of techniques will elucidate the proper characterization of the network including the whole array of inhibitory subtype cells. Behaviorally, and in females, impairment of ASA was observed when other brain areas in addition to NCM may have been affected by systemic FAD. The transient periods of low circulating estrogen caused by FAD could resemble the periods of estrogen fluctuations typically observed during human perimenopause in which ovarian function gradually decrease until continuously low estrogen levels at menopause (Brooks et al., 2016). This could be a factor responsible for the diminished ability that some middle-age women have to successfully recognize and temporally process speech-in-noise (Helfer and Vargo, 2009; Hederstierna et al., 2010; Helfer and Jesse, 2020). It would also explain why hormone replacement therapy improves speech-in-noise detection and protects against hearing loss (Helfer, 2004; Frisina and Frisina, 2016; Williamson et al., 2019). Moreover, this work can support the growing concern about the unfavorable cognitive effects that aromatase inhibitors, commonly used as anti-cancer treatment, can have in recovering patients (Hurria et al., 2014; Blaustein, 2017).

## Synthesis

Reviewing Editor: Julie Bakker, University of Liege Decisions are customarily a result of the Reviewing Editor and the peer reviewers coming together and discussing their recommendations until a consensus is reached. When revisions are invited, a fact-based synthesis statement explaining their decision and outlining what is needed to prepare a revision will be listed below. The following reviewer(s) agreed to reveal their identity: Jacques Balthazart.

Synthesis: the paper has been reviewed by only one reviewer, but I have also read the paper since we had trouble getting a second reviewer and didn't want to delay the reviewing process too much. I agree with the comments and suggestions made by the reviewer.

Reviewer 1

This paper describes two sets of experiments analyzing the effects of aromatase inhibition by administration of the selective inhibitor Fadrozole (FAD) on the detection of conspecific songs in the absence and in the presence of increasing sounds distractors, a task know as auditory scene analysis (ASA). FAD was either administered centrally in NCM or given orally and effects were analyzed either by electrophysiology of NCM neurons or behaviorally via an operant conditioning procedure.

FAD retrodialyzed in NCM decreased responsiveness and timing reliability on narrow spiking (presumably inhibitory) neurons but not of broad spiking neurons. At the same time FAD decreased aspects of song detection by neurons in face of confounding stimuli increasing in amplitude with some of these effects being more prominent in females.

When tested at the behavioral level via operant conditioning, oral (systemic) FAD administration transiently (for the first 2 hours) decreased correct choices of the target stimulus in both sexes but surprisingly increased performance in the ASA in females but not in males. In contrast FAD administration directly in NCM had no detectable impact on the bird's behavioral performance.

This is an excellent set of studies in the tradition of work from the Remage-Healey lab. Studies were performed with very sophisticated methods that were previously validated or are direct extensions of methods developed in the host lab. Overall the presentation of results is very clear even if several aspects are unexpected and actually counterintuitive. The discussion however presents the most likely explanations in particular for the unanticipated aspects. As a consequence, I have no major concern about this fine study.

The only general point that could be raised is that given the limited number of subjects in some experiments, some conclusions are associated with a limited power. As a partial fix for this potential problem, most graphs nicely present all individual data so that the reader will be able to assess the individual reproducibility of results. In some cases, this individual variability is high which may have prevented statistical significance of differences. This problem might actually be more prominent for some of the male-female comparisons. At one point (line 714) the authors discuss the low power of previous studies on sex differences; it might be useful to mention that this limitation also applies to aspects of the present study in the discussion in addition to the other explanations that are presented and make a lot of sense.

Also, for very understandable reasons, units that were analyzed in detail were selected based on several criteria (Line 241: out of noisy clusters, high signal to noise ratio, low overlap, etc). How can you be sure that the selected units are really representative on the entire electrical activity?

Besides these two general comments I only have a few minor suggestions or suggested corrections.

Line 427: the word "data" or similar is missing after "Behavioral"

Line 526: the probability here should be 0.06 according to the figure 6a. Please reconcile the two values.

Line 697-697: it seems to me that "the regeneration of aromatase..." would be too slow to explain any aspect of the present results. This should be either explained in more detail or deleted.

Line 817 and 73 : the 1st author in this reference is "de Bournonville" not "Bournonville" and the reference should be moved at the letter D in the list.
